# Supplementary material for: Answers to the Frequently Asked Questions Regarding Horse Feeding and Management Practices to Reduce the Risk of Atypical Myopathy
Source: Animals (Basel). 2020 Feb 24;10(2):365. doi: 10.3390/ani10020365 (PMC7071031; doi:10.3390/ani10020365)
Supplement: Supplementary file 1 [file animals-10-00365-s001.pdf]

animals

MDPI

French translation of the article:

Votion D-M., François A-C., Kruse C., Renaud R., Farinelle A., Bouquieaux M-C., Marcillaud-Pitel C., Gustin P. Answers to the frequently asked questions regarding horse feeding and management practices to reduce the risk of atypical myopathy.

Réponses aux Questions Fréquemment Posées (FAQs) à Propos de l'Alimentation et de la Gestion des Équidés ainsi que de la gestion des Pâtures afin de Réduire le Risque de Myopathie Atypique

Traduction et illustration : Anne-Christine François[1] & Dominique-Marie Votion[2,*]

[1] Département des Sciences Fonctionnelles, Faculté de Médecine vétérinaire, Pharmacologie et Toxicologie, Fundamental and Applied Research for Animals & Health (FARAH), Université de Liège, 4000 Liège 1 (Sart Tilman), Belgique; acfrancois@uliege.be

[2] Pole Équin, Fundamental and Applied Research for Animals & Health (FARAH), Faculté de Médecine vétérinaire, Université de Liège, 4000 Liège 1 (Sart Tilman), Belgique

* Correspondance: dominique.votion@uliege.be

Résumé : La myopathie atypique des équidés est une intoxication sévère due à l'ingestion des samares et des plantules de certaines espèces d'érables (*Acer* spp.). L'érable sycomore (*Acer pseudoplatanus*) est impliqué dans les cas européens de myopathie atypique tandis que l'érable negundo (*Acer negundo*) est reconnu comme étant la cause de cette maladie aux États-Unis d'Amérique. En Europe, les animaux jeunes et inactifs avec un score corporel qualifié de normal à mince et ne recevant pas d'alimentation supplémentaire, excepté du foin en automne, ont statistiquement plus de risque de développer la maladie. Ce risque élevé est aussi associé au pâturage permanent et à un environnement humide. En effet, les feuilles mortes accumulées en automne et la présence d'arbres ou de bois exposent vraisemblablement les chevaux aux samares de sycomore. Ce manuscrit répond aux questions les plus fréquemment posées par la filière équine à propos de l'alimentation et de la gestion des chevaux ainsi que des pâtures afin de réduire le risque de myopathie atypique. Les réponses sont basées sur la collecte de données depuis 2006 jusque 2019 par l'« Atypical Myopathy Alert Group » (AMAG, Belgium) et le « Réseau d'épidémio-surveillance en Pathologie équine » (RESPE, France) ainsi que sur la revue de la littérature scientifique.

Résumé: En 2014, la cause de la myopathie atypique (MA) a été identifiée : il s'agit de l'*Acer pseudoplatanus* (érable sycomore) en Europe. L'émergence de cette intoxication saisonnière causée par un arbre « indigène » a soulevé nombre d'interrogations. Ce manuscrit a pour but de répondre au cinq questions les plus fréquemment posées à propos (1) de l'indentification de l'érable sycomore, de la réduction du risque au niveau (2) de la pâture et (3) des équidés, (4) du risque associé avec des pâtures comportant des érables sycomores et qui ont toujours accueilli des chevaux sans que ceux-ci ne soient touchés par cette maladie et (5) la durée des périodes à risque. Les réponses ont été formulées à partir d'une revue de la littérature scientifique et de la collecte de données par les réseaux de surveillance de la MA. Un guide est proposé pour différencier les différentes espèces d'érables (FAQ1). Afin de réduire le risque lié au pâturage, il est conseillé d'éviter les prairies humides, le pâturage permanent, l'épandage de fumier sur les pâtures avec des érables sycomores à proximité et la présence de ces érables autour des pâturages (FAQ2). Quant au risque lié à l'équidé lui-même, il est conseillé de réduire le temps de pâture selon les conditions météorologiques et à moins de six heures par jour durant les périodes à risques pour les chevaux séjournant sur des pâtures à risque, de fournir de l'alimentation en supplément comme du foin non

contaminé, de fournir de l'eau issue du réseau de distribution ainsi que des vitamines et un bloc de sel (FAQ3). Toutes les pâtures avec des érables sycomores à proximité sont des prairies à risque (FAQ4). Enfin, 94% des cas déclarés de MA se situent dans une période de 3 mois commençant en octobre et en mars suite à l'ingestion de samares ou de plantules respectivement (FAQ5).

Mots clés : myopathie atypique des équidés; *Acer* spp.; facteurs de risque; environnement

1. Introduction

La myopathie atypique des équidés (MA) est une intoxication sévère liée au pâturage qui se déclare en automne et au printemps suite à l'ingestion de graines et de plantules de certaines espèces d'érable (*Acer*) respectivement. Cette intoxication environnementale est causée par l'*Acer pseudoplatanus* (érable sycomore ; Figure 1) en Europe et l'*Acer negundo* (érable négundo ; Figure 2) aux USA [1,2]. Ces arbres contiennent plusieurs toxines [3]. L'ingestion des graines appelées samares (Figure 3) ou des plantules (Figure 4) des arbres incriminés s'accompagne de l'ingestion de deux acides cyclopropyle-amines : l'hypoglycine A (HGA) et la méthylène-cyclopropyle-glycine (MCPG) [4]. L'implication de ces toxines dans les cas européens de MA est confirmée. Longtemps avant la découverte de la cause de la MA, Fowden and Pratt (1973) [3], ont montré la présence de ces toxines dans les samares de différents représentants de la famille des érables. Ainsi, de l'HGA et du MCPG ont été mis en évidence dans les graines de l'*Acer pseudoplatanus* et de l'*Acer negundo*. Au contraire, d'autres érables communément trouvés en Europe comme l'érable plane (*Acer platanoides* ; Figure 5) ou l'érable champêtre (*Acer campestre* ; Figure 6) ne contiennent pas ces composés [3].

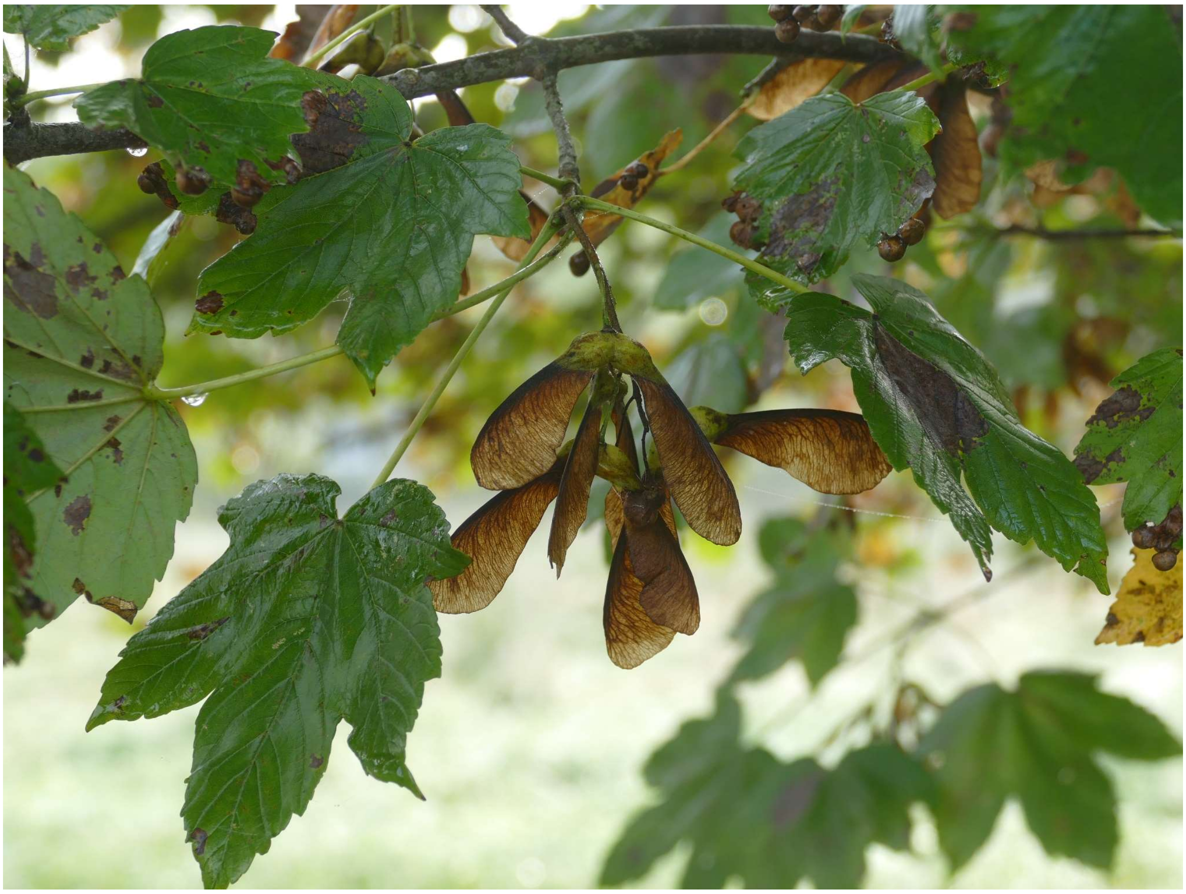

Figure 1. *Acer pseudoplatanus* (érable sycomore)
La feuille d'érable sycomore comporte 5 lobes à bords dentés avec des sinus aigus. Les samares sont portées par paire formant un angle aigu. Crédit photo : D-M. Votion

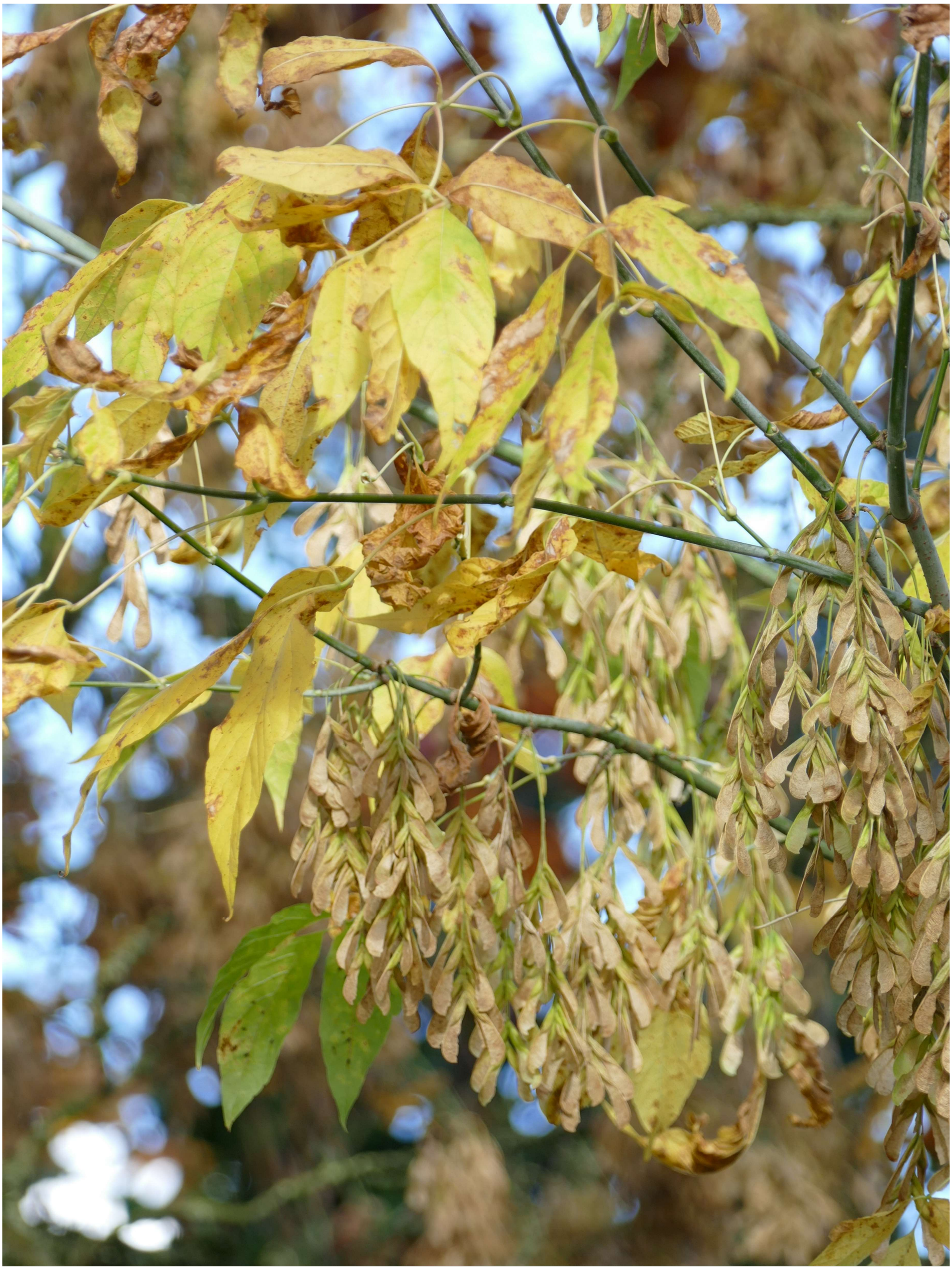

Figure 2. *Acer negundo* (érable négundo)
Le feuillage de cet érable est particulier car les feuilles sont pennées avec 3 à 7 folioles (c'est pourquoi il est aussi appelé parfois érable à feuilles de frêne). Crédit photo : D-M. Votion

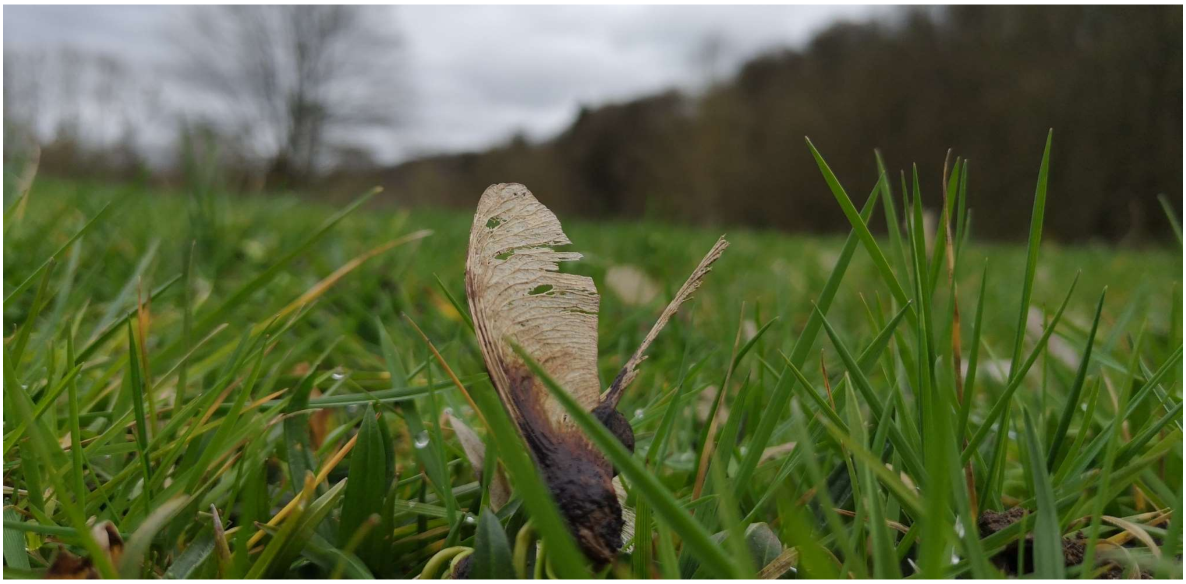

Figure 3. Samares de l'*Acer pseudoplatanus* (érable sycomore)
Les samares de l'érable sycomore tombées au sol, à l'automne, sont la source toxique responsable de la myopathie atypique. Crédit photo : D-M. Votion

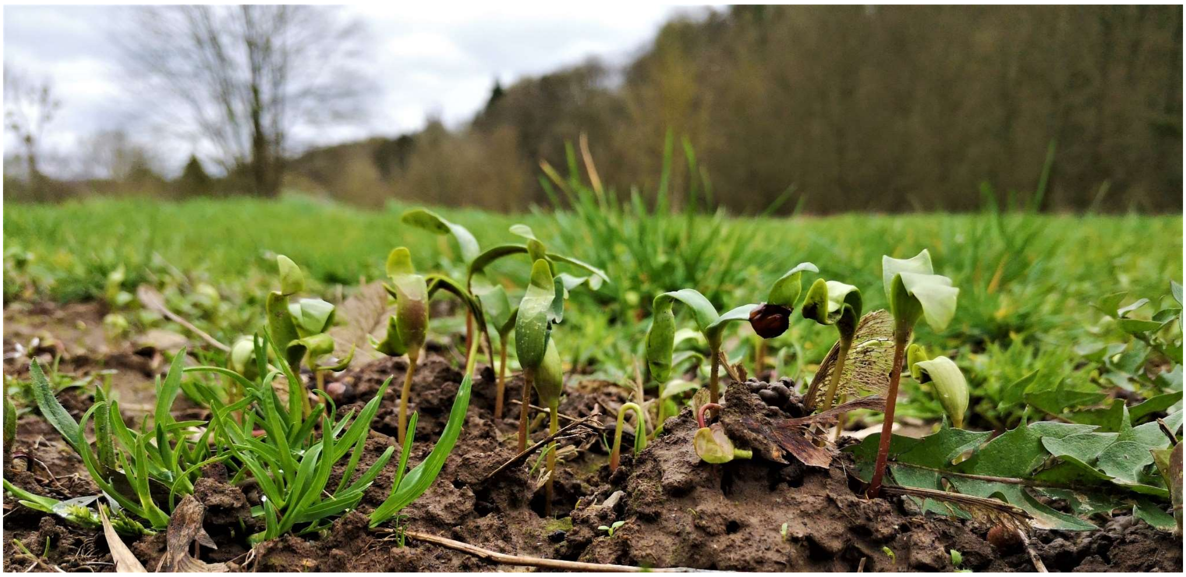

Figure 4. Plantules de l'*Acer pseudoplatanus* (érable sycomore)
Les plantules de l'érable sycomore émergeant au printemps sont la source toxique responsable de la myopathie atypique. Crédit photo : D-M. Votion

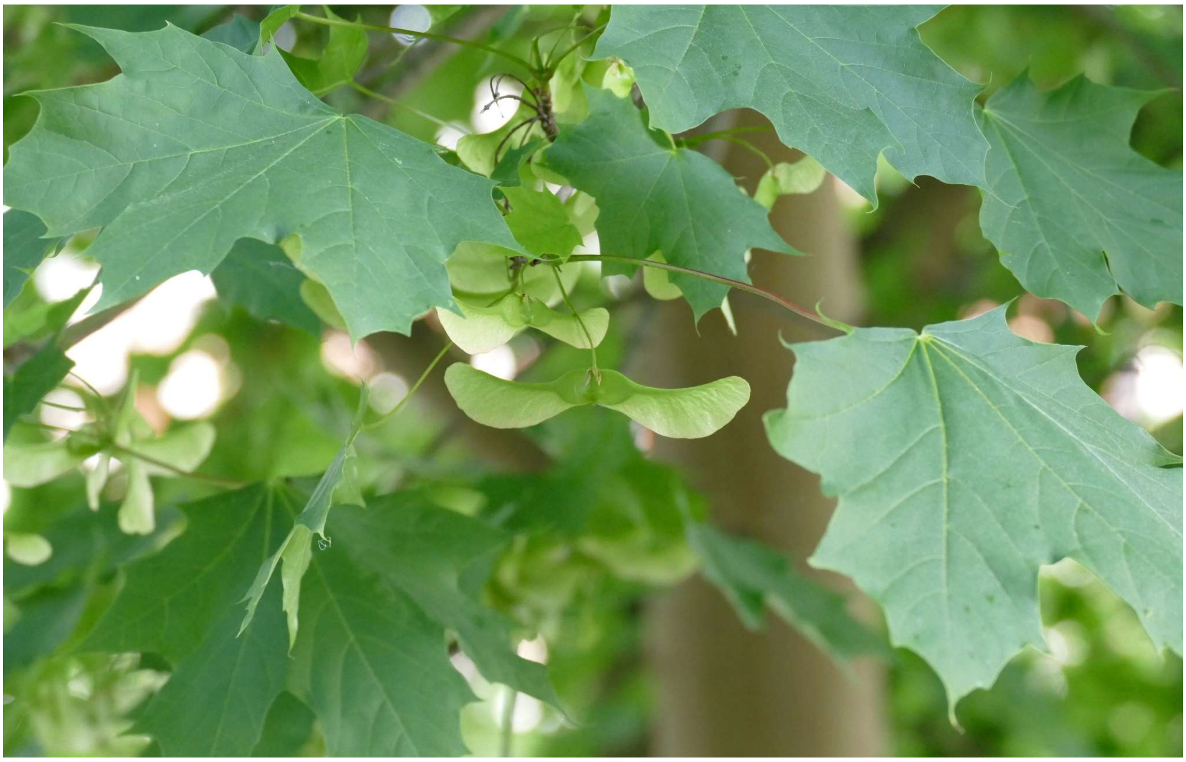

Figure 5. *Acer platanoides* (érable plane)
Les feuilles de l'érables planes ont 5 lobes acuminés (i.e., « pointus ») avec des sinus obtus. Les samares sont portées par paire formant un angle obtus. Crédit photo : D-M. Votion

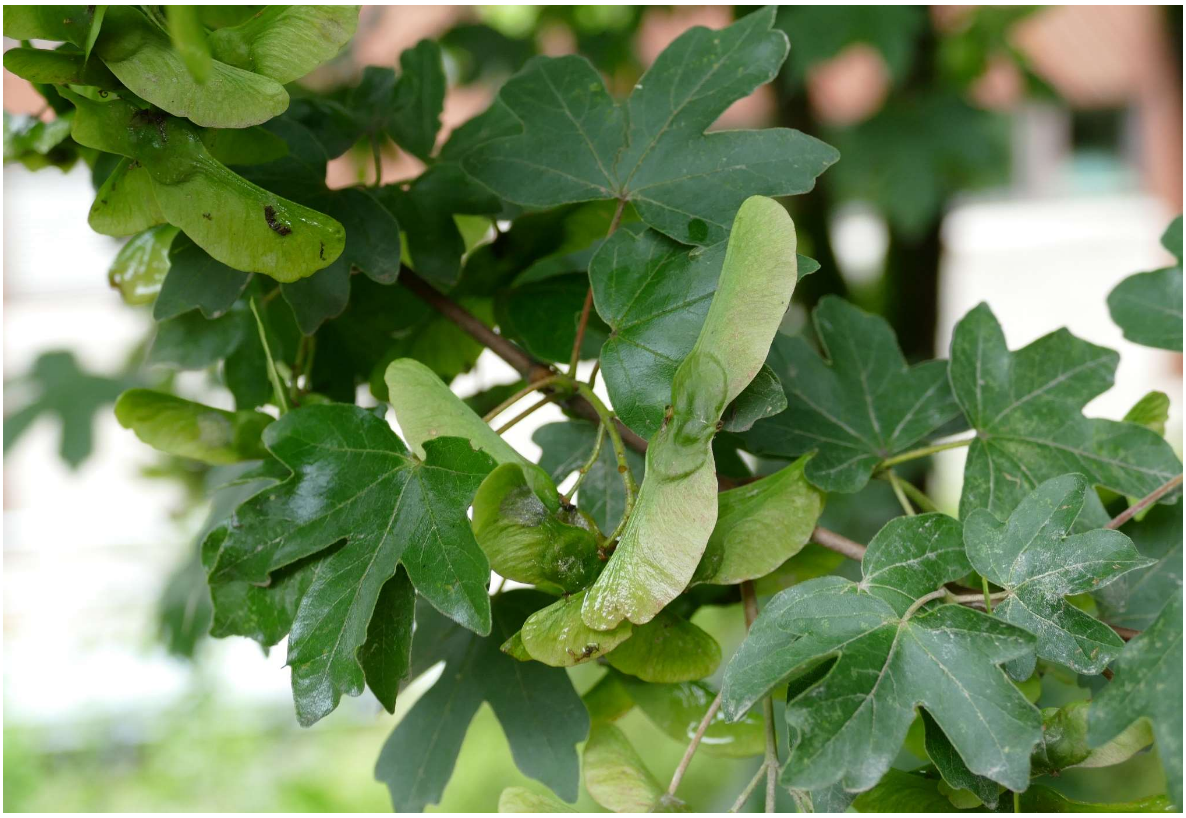

Figure 6. *Acer campestre* (érable champêtre)
Les feuilles de l'érables champêtre ont 5 lobes lisses avec des sinus aigus. Elles sont plus petites que les feuilles des érables sycomores et planes. Les samares sont portées par paire formant un angle plat. Crédit photo : D-M. Votion

La toxicité de l'HGA et du MCPG n'est pas due à ces molécules par elles-mêmes mais par leurs métabolites toxiques : respectivement, le méthylènecyclopropylacetyl-CoA (MCPA-CoA) et le méthylènecyclopropylformyl-CoA (MCPF-CoA) [4,5]. Ceux-ci inhibent certaines enzymes responsables de la β-oxydation des acides gras et donc la production d'énergie *via* le métabolisme des lipides [5,6]. Les signes cliniques de cette intoxication sont un syndrome de rhabdomyolyse aigue non corrélée avec la réalisation d'un effort physique. Cette image clinique peut être observée chez plusieurs chevaux du même groupe [7–10]. Dans plus de 50% des cas, les signes cliniques suivants sont observés : faiblesse, décubitus, myoglobinurie, vessie distendue, tremblements ou fasciculation musculaires, réticence à se mouvoir, transpiration, normothermie et muqueuses congestionnées [10,11,12]. La MA a un haut taux de mortalité (i.e., 74%) qui varie selon les pays et les années (de 43% [11] à 97% [13]). Ce taux de mortalité général de 74% représente une moyenne à travers les pays inclus dans l'étude de van Galen et al., (2012) et ne prend pas en compte les différentes sources de variabilité [11]. Par exemple, une étude rapporte un taux de mortalité plus faible (i.e., 44%) chez les chevaux hospitalisés [14]. L'hypothèse pour expliquer cette différence est que les animaux conduit jusqu'à un hôpital sont ceux dont la symptomatologie est la moins sévère et que des traitements symptomatiques plus appropriés peuvent être plus facilement administrés en clinique que sur le terrain. Ces deux facteurs contribueraient à expliquer le taux de survie plus important des équidés hospitalisés que ceux restés au pré. Dans tous les cas, seule l'administration de vitamines et d'antioxydants s'est avérée bénéfique pour la survie des cas de MA [12,15].

En 2004, un groupe nommé « Atypical Myopathy Alert Group » (AMAG) a été créé afin d'alerter les praticiens équins et les propriétaires de chevaux sur les périodes les plus à risque de MA. Les alertes sont publiées suite à la déclaration de cas auprès de l'AMAG qui se charge de mettre à jour régulièrement le dénombrement des cas. En plus de son rôle de surveillance, l'AMAG collecte des données épidémiologiques depuis 2006 à l'échelle de l'Europe. Les cas français sont collectés en étroite collaboration avec le Réseau d'Épidémio-Surveillance en Pathologie Équine (RESPE) qui surveille les maladies touchant les chevaux au travers d'un réseau de vétérinaires sentinelles. Grâce à cette surveillance au niveau européen, il a été possible de découvrir que plusieurs cerfs du Père David (*Elaphurus davidianus*) ont succombé de cette même intoxication dans plusieurs zoos en Allemagne. Cette observation suggère que les ruminants pâturant à proximité d'érable sycomore pourraient aussi être intoxiqués [16].

Au regard du taux élevé de mortalité et l'absence de traitement spécifique, la prévention est la clé pour empêcher les animaux de développer cette maladie. Avant de découvrir la cause de la MA, les études épidémiologiques ont révélé certains facteurs de risque associés à la gestion des chevaux ou des pâtures [15,17]. En 2014, la cause de la MA a été découverte et reliée à l'*Acer pseudoplatanus* en Europe [1]. Malgré la connaissance de la cause de la MA, les séries de cas cliniques ont continué à se produire. Une telle maladie causée par un arbre considéré comme indigène a suscité beaucoup d'interrogation de la filière équine.

La question la plus fréquemment posée concerne l'identification des arbres toxiques. Les autres questions s'intéressent à l'alimentation et à la gestion des chevaux afin de réduire le risque d'intoxication. Ces principales questions peuvent être résumées comme suit : (FAQ1) « Quels sont les arbres toxiques ? » ; « Est-ce que cet arbre est un sycomore? Si oui, est-il toxique ? » ; (FAQ2) « Comment la MA peut-elle être prévenue (au niveau de la pâture) ? » ; (FAQ3) "Comment la MA peut-elle être prévenue (au niveau du cheval)?"; (FAQ4) « Notre pâture est entourée d'érables sycomores mais il n'y a jamais eu de cas de MA avant. Est-ce que cela signifie que notre équidé est en sécurité dans cette pâture ? » et (FAQ5), « Quand le risque de MA commence-t-il et quand se termine-t-il en automne et au printemps ? ».

Cet article répond à ces questions fréquemment posées (FAQs) touchant la nutrition et la gestion des animaux afin de prévenir la MA en se basant sur la littérature scientifique et en analysant les données épidémiologiques collectées depuis 2006.

2. Matériel et méthode

2.1. Revue de la littérature

Une revue systématique de la littérature a été menée en utilisant les mots clés "atypical myopathy" et "horse" à l'aide des bases de données PubMed et Scopus. Les résumés de minutes de congrès dédiés aux chevaux ont aussi été consultés (e.g., AAEP—American Association of Equine Practitioners Annual Convention, AVEF—Association des Vétérinaires Équins Français, BEPS—Belgian Equine Practitioners Society Study Days, BEVA—British Equine Veterinary Association and, WEVA—World Equine Veterinary Association Congress).

2.2. Données épidémiologiques

Les informations concernant les cas européens de MA sur une période de 13 ans (2006-2019) ont été collectées grâce à des questionnaires standardisés disponibles sur les sites de l'AMAG (http://www.myopathie-atypique.be) et du RESPE (https://respe.net). Ces formulaires ont été complétés par e-mail ou par téléphone avec les propriétaires ou les vétérinaires quand cela était possible. Certaines informations ont été recueillies directement par téléphone ou par mail grâce à un contact entre le propriétaire/vétérinaire et le chercheur principal de cette étude. Les informations à propos de la gestion et de l'environnement du cheval malade ont été obtenues via le propriétaire tandis que les données cliniques ont été récoltées auprès du vétérinaire. Les cas cliniques se déroulant entre le 1er septembre et la fin février ont été classés en « cas automnaux » et ceux entre le 1er mars et la fin aout, ont été définis comme des « cas de printemps ».

3. Résultats

3.1. Revue de la littérature

Parmi les 68 résultats fournis par PubMed, cinq ont été rejetés car ils ne concernaient pas la MA. Quant à la recherche sur Scopus, 514 résultats ont été obtenus et ont été affinés en sélectionnant uniquement les articles de recherche. Sur les 82 études restantes, 63 étaient hors sujet et ont donc été éliminées. Au total, plus de 127 documents (articles de recherche et résumés) ont été considérés.

3.2. Données Epidémiologiques

Les données épidémiologiques des 3039 cas ont été enregistrées dans 14 pays différents depuis l'automne 2006 au 30 novembre 2019 (Figure 7, Table 1). Cet ensemble de données inclut tous les cas communiqués aux réseaux de surveillance avec un diagnostic provisoire de MA. Durant cette période, 14 séries cliniques automnales ont été enregistrées avec une moyenne (± S.D.) de 164.6 ± 153.3 (médiane 79) cas rapportés d'une part. D'autre part, 12 séries printanières ont été enregistrées avec une moyenne (± S.D.) de 56.5 ± 77.3 (médiane 24) chevaux malades par série clinique. Pour toutes les séries cliniques, la moyenne (± S.D.) du nombre de cas est de 112.5 ± 132.4 (médiane 41). Une partie de ces données (n = 824) a été analysée précédemment pour définir les facteurs de risque (Table 2) et de protection (Table 3) sur des cas survenus en Belgique [17], au Royaume-Uni [12] et à un niveau européen [11,15]. Les chevaux particulièrement à risque de développer la MA sont les chevaux jeunes (i.e., âgés de moins de 3 ans) et inactifs avec un score corporel de normal à mince et recevant du foin en automne [17]. Le risque d'intoxication est aussi associé au pâturage permanent dans un environnement humide où les feuilles mortes s'accumulent en automne avec la présence d'arbres ou de bois et donc probablement exposé aux érables incriminés [12,15,17]. Dix années se sont écoulées depuis la réalisation de la dernière étude épidémiologique européenne [11,15], 2433 nouveaux cas ont été enregistrés, ce qui permet d'atteindre un nombre total de cas disponibles pour cette étude de 3039. L'entièreté de la base de données (i.e., 2006-2019) a été parcourue afin de retirer les chevaux qui n'étaient pas en prairie lors du déclenchement des symptômes ou qui n'étaient pas au pré la semaine précédant les signes cliniques, les chevaux dont le diagnostic final était une autre maladie que la MA ainsi que les chevaux avec une faible probabilité d'intoxication (e.g., pas de pigmenturie et une

activité sérique des créatines kinases <10.000 UI/L ; valeurs normales 50–200: UI/L) en accord avec van Galen et al., 2012 [11]. Contrairement à l'étude de van Galen et al., 2012 [11], les cas insuffisamment documentés pour permettre la pose d'un diagnostic final n'ont pas été retirés de notre étude. Un total de 2371 cas sont donc inclus dans cette étude. La distribution de l'âges de tous les cas inclus est représentée dans la Figure 8. L'occurrence hebdomadaire des cas durant l'automne et le printemps est représentée dans les Figures 9a,b respectivement.

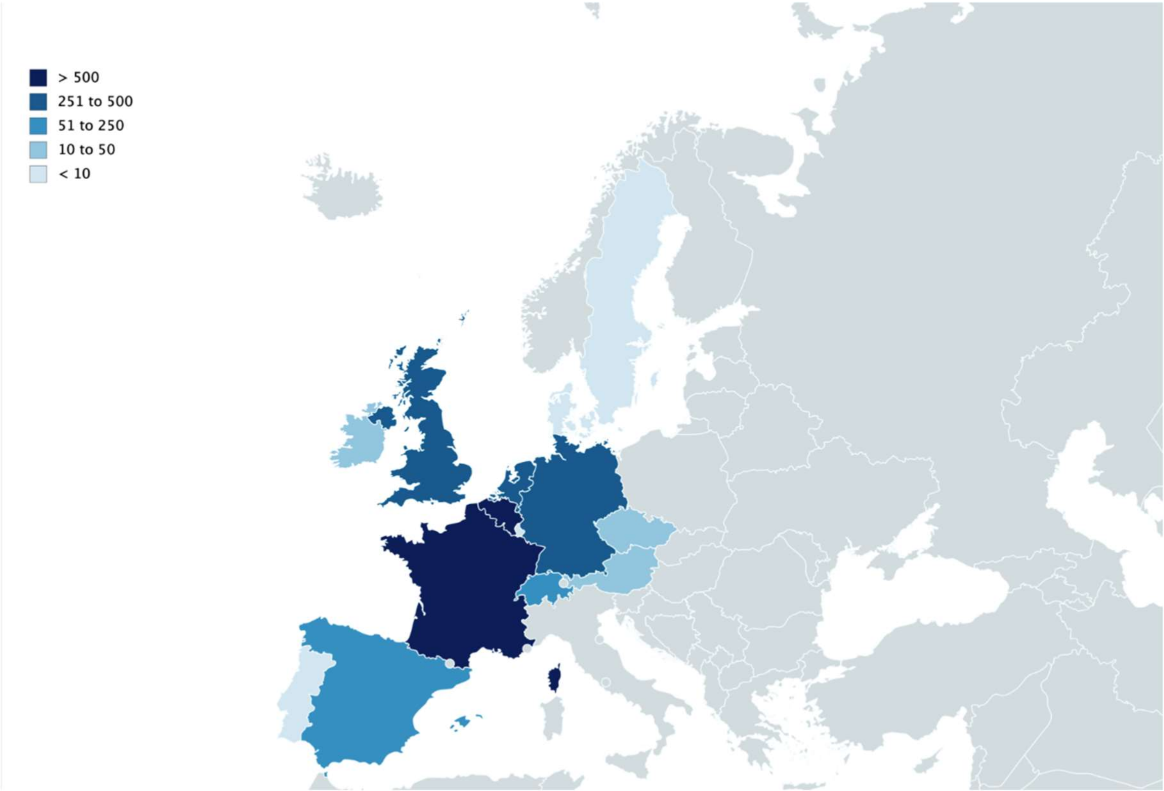

Figure 7. Distribution des cas européens de myopathie atypique notifiés aux réseaux de surveillance depuis l'automne 2006 jusqu'à novembre 2019.

Table 1. Nombre total de cas de myopathie atypique en Europe notifiés aux réseaux de surveillance depuis l'automne 2006 jusqu'à novembre 2019 (n = 3039).

**Année**	**2006**	**2007**		**2008**		**2009**		**2010**		**2011**		**2012**		**2013**		**2014 ***		**2015**		**2016**		**2017**		**2018**		**2019**		
**Pays**	**Automne**	**Printemps**	**Automne**	**Printemps**	**Automne**	**Printemps**	**Automne**	**Printemps**	**Automne**	**Printemps**	**Automne**	**Printemps**	**Automne**	**Printemps**	**Automne**	**Printemps**	**Automne**	**Printemps**	**Automne**	**Printemps**	**Automne**	**Printemps**	**Automne**	**Printemps**	**Automne**	**Printemps**	**Automne**	**Total/pays**
Autriche	0	0	0	0	0	0	0	0	0	0	4	0	0	0	0	0	0	0	0	0	0	0	0	4	9	0	0	**17**
Belgique	46	7	18	0	5	0	69	13	14	3	18	0	1	0	141	8	51	5	6	8	52	20	5	1	191	49	1	**732**
Rép. Tchèque	0	0	0	0	0	0	0	0	0	0	0	0	0	0	9	0	0	0	0	0	2	17	0	0	19	2	0	**49**
Danemark	2	0	0	0	0	0	2	0	0	0	0	0	0	0	0	0	0	0	2	0	0	0	0	0	2	0	0	**8**
France	32	1	11	18	11	0	134	106	32	10	40	16	4	1	64	13	71	9	24	8	194	181	31	18	114	47	26	**1216**
Allemagne	7	0	3	5	0	0	93	21	2	2	59	2	0	0	24	1	8	0	0	0	21	20	4	0	33	4	2	**311**
Irlande	0	0	0	0	0	0	2	0	0	0	0	0	0	0	2	0	38	0	0	0	1	2	0	0	1	0	0	**46**
Luxembourg	1	0	0	0	0	0	2	0	0	0	0	0	0	0	0	0	0	0	0	0	0	0	0	0	0	0	0	**3**
Portugal	0	0	0	0	0	0	0	0	0	1	0	0	0	0	0	0	0	0	0	0	1	0	0	0	0	0	0	**2**
Espagne	0	0	0	0	0	0	0	0	0	0	52	0	0	0	0	0	0	0	0	0	0	0	0	0	0	0	0	**52**
Suède	0	0	0	0	0	0	0	1	0	0	1	0	0	0	0	5	0	0	0	0	0	0	0	0	0	0	0	**7**
Suisse	0	0	9	0	0	0	31	3	0	0	6	0	0	1	12	0	0	0	0	0	9	3	0	0	7	0	0	**81**
Pays-Bas	13	0	3	0	2	0	34	7	0	0	0	0	0	0	18	1	4	0	0	0	1	2	0	2	9	10	1	**107**
Royaume-Uni	1	0	13	0	0	0	39	20	3	0	33	6	2	2	52	13	154	20	2	1	11	11	3	1	13	3	5	**408**
**Total/saison**	**102**	**8**	**57**	**23**	**18**	**0**	**406**	**171**	**51**	**16**	**213**	**24**	**7**	**4**	**322**	**41**	**326**	**34**	**34**	**17**	**292**	**256**	**43**	**26**	**398**	**115**	**35**	**3039**
**Total/année**	**102**	**65**		**41**		**406**		**222**		**229**		**31**		**326**		**367**		**68**		**309**		**299**		**424**		**150**		

Commentaires: Ce dernier comptage remplace les données précédemment publiées; le mot « automne » ne doit pas être pris *sensus stricto* car les séries de cas cliniques continuent en hiver; (*) in 2014, découverte de la cause de la myopathie atypique en Europe : *Acer pseudoplatanus* (érable sycomore).

Table 2. Facteurs de risque identifiés dans de précédentes études épidémiologiques [12,15,17]

Catégorie	Facteurs de risque	Rapport de cotes	95% Cl pour les rapport de cotes
Données démographiques	Jeunes chevaux (< 3 ans)		
	Mince<br>(ou score corporel normal*)	3.08 [17](b)<br>(3.85 [15] /2.20 [17](b))	1.01–9.39 [17] (b)<br>(1.77–8.37 [15] /1.01–4.79 [17] (b))
Gestion du cheval	Au pré 24/24h toute l'année	5.42 [15]<br>3.07 [17](b) en hiver<br>3.78 [17](b) au printemps<br>23.2 [17](b) en été<br>10.9[17)(b) en automne	2.577-11.42 [15]<br>1.45-6.50 [17] (b) en hiver<br>1.49-9.59 [17] (b) au printemps<br>1.41-382 [17] (b) en été<br>3.56-33.4 [17] (b) en automne
	Pas actif physiquement	11.8 [17](b)	5.02–27.8 [17] (b)
Alimentation	Foin donné en automne	4.09 [17](a)	1.18–14.1 [17] (a)
Pâture	Historique de précédents décès de chevaux sur la pâture	4.45 [17](b)	1.61–12.29 [17] (b)
	Pâturage luxuriant en hiver	3.95 [17](b)	1.49–10.46 [17] (b)
	Pâturage en pente / pente raide	3.43 [15]<br>3.70 [17](b)	1.52–7.77 [15]<br>1.58–8.68 [17] (b)
	Accès à des feuilles mortes empilées en automne	11.11 [15]<br>10.47 [17](b)	4.82–25.59 [15]<br>2.82–40.88 [17] (b)
	Présence d'arbre dans la pâture *	7,82 [15]	1.99–30.73 [15]
	Bois morts dans la pâture	3.12 [15]	1.42–6.84 [15]
	Prairie humide	2.63 [17](b)	1.29–5.36 [17] (b)
	Praire entourée ou contenant un cours d'eau/ une rivière	2.78 [17](b)	1.24–6.19 [17] (b)
	Épandage de fumier	5.73 [17](b)	2.40–13.69 [17] (b)

CI = intervalle de confiance ; (*) Paramètres inconstants selon les études, [15] cas avec une forte probabilité de myopathie atypique confirmée *vs.* cas avec une faible probabilité d'avoir une myopathie atypique ou avec un autre diagnostic, [17] (a) cas confirmés *vs.* co-pâturants cliniquement sains, [17] (b) cas confirmés *vs.* cas contrôles

Table 3. Facteurs de protection identifiés dans de précédentes études épidémiologiques [12,15,17]

Catégorie	Facteurs de protection	Rapport de cotes	95% Cl pour les rapport de cotes
**Données démographiques**	Surpoids	0.25 [17](b)	0.09-0.69 [17](b)
**Gestion du cheval**	Vermifugation fréquente	0.11 [17](a)<br>0.05 [17](b)	0.01-0.67 [17](a)<br>0.01-0.16 [17](b)
	Vaccination fréquente	0.10 [17](b)	0.05-0.21 [17](b)
	Activité physique régulière	0.08 [17]	0.03-0.19 [17]
	Temps de pâturage en fonction des conditions météorologiques au printemps et en automne	0.24 printemps [17]<br>0.10 automne [17]	0.06-0.89 printemps [17]<br>0.02-0.56 automne [17]
	< 6h au pré par jour	0.04 [15]<br>0.62 [17]	0.01-0.19 [15]<br>0.16-2.36 [17]
	Pas d'accès à la pâture	0.03 [15]	0.00-0.22 [15]
**Alimentation Approvisionnement en eau**	Alimentation supplémentaire toute l'année	0.17 [15]	0.05-0.59 [15]
	Ensilage et aliments concentrés en automne + maïs en hiver	0.20 [17](a) pour l'ensilage<br>0.19 [17](a) pour l'alimentation concentrée<br>0.22 [17](a) pour le maïs	0.04-0.94 [17](a) pour l'ensilage<br>0.04-0.87 [17](a) pour l'alimentation concentrée<br>0.05-0.93 [17](a) pour le maïs
	Bloc de sel (toute l'année)	3.52 [12]<br>0.20 [17]	1.08-11.47 [12]<br>0.09-0.40 [17]
	Alimentation en eau dans un réservoir / une baignoire	0.25 [15]	0.09-0.69 [15]
	Eau fournie par le réseau de distribution	0.39 [17](b)	0.17-0.88 [17](b)
**Pâture**	Pente douce	0.34 [17](b)	0.14-0.84 [17](b)

CI = intervalle de confiance ; [12] survivants *vs*. non-survivants ; [15] cas avec une forte probabilité de myopathie atypique confirmée *vs*. cas avec une faible probabilité d'avoir une myopathie atypique ou avec un autre diagnostic ; [17] (a) cas confirmés *vs*. co-pâturants cliniquement sains ; [17] (b) cas confirmés *vs*. cas contrôles

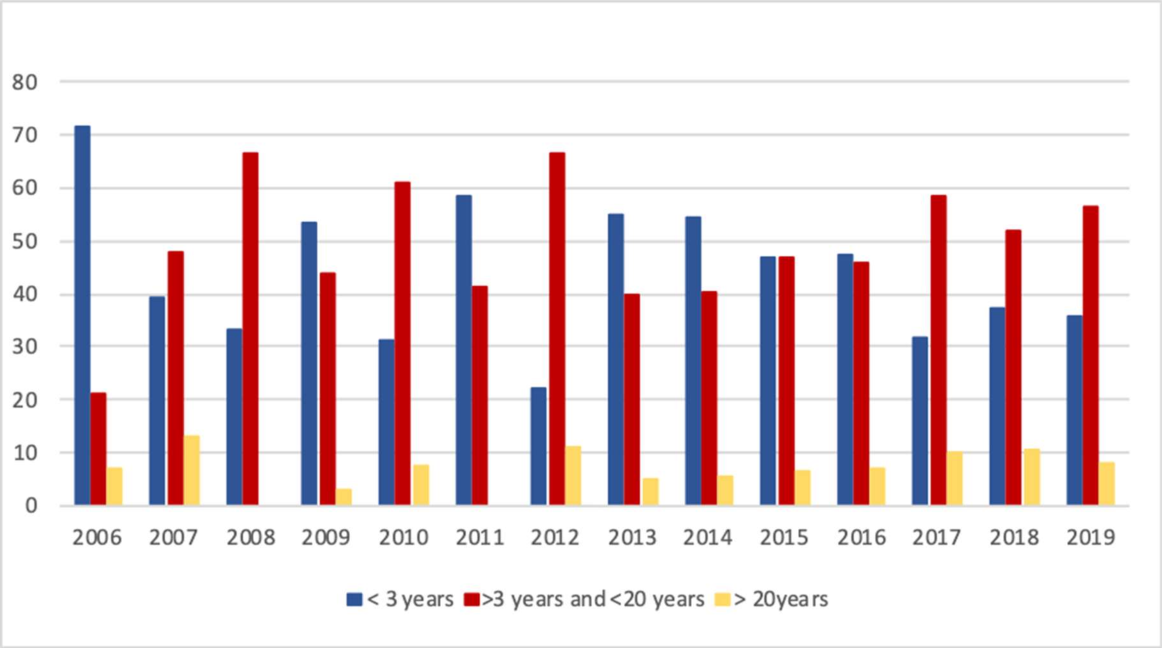

Figure 8. Distribution de la fréquence des équidés selon leur catégorie d'âge : <3 ans, >3 ans et <20 ans et >20 ans (n = 1510) sur toute la durée de l'étude (2006–2019).

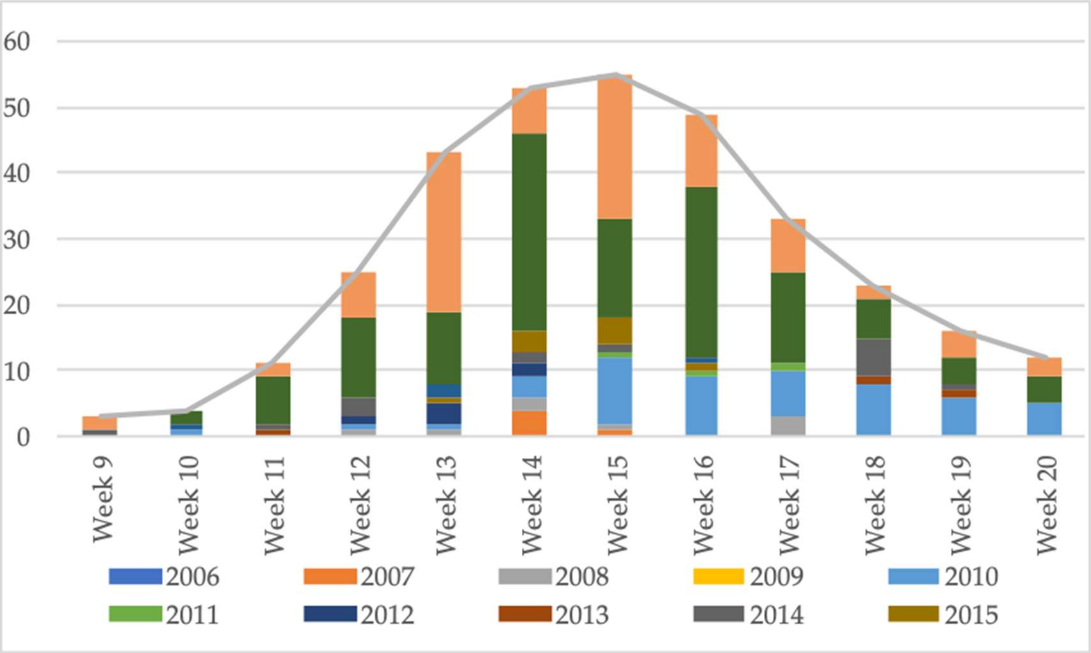

(**a**)

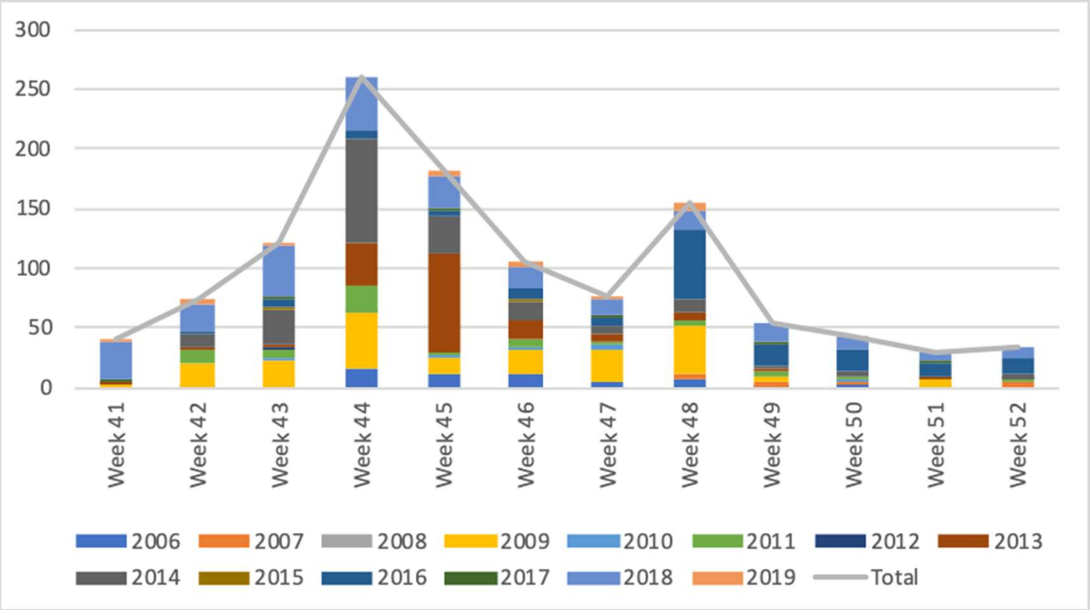

(**b**)

Figure 9. (**a**). Cas de printemps : occurrence hebdomadaire de myopathie atypique depuis la semaine 9 (1er mars) jusqu'à la semaine 20 (31 mai) sur toute la durée de l'étude (2006–2019); (**b**). Cas d'automne: occurrence hebdomadaire de myopathie atypique depuis la semaine 41 (1er octobre) jusqu'à la semaine 52 (31 Décembre) sur toute la durée de l'étude (2006–2019).

4. Discussion

Les données exploitées proviennent de précédentes études épidémiologiques complétées par l'ajout de nouvelles données. Les résultats contribuent à répondre aux FAQs concernant l'alimentation et les pratiques de gestion afin de réduire le risque de MA.

4.1. FAQ1: "Quels sont les arbres toxiques ? Est-ce que cet arbre est un sycomore? Si oui, est-il toxique? »

Cette question concernant quels arbres sont en réalité toxiques est souvent liée à une demande d'identification des arbres sur la pâture. La consultation de la base de données indique que 99% des pâtures contiennent ou sont directement bordées par des arbres. Cependant, en regardant les données depuis 2014 jusqu'à maintenant, 20% (92/456) des propriétaires de chevaux atteints de MA ne peuvent pas préciser si des samares ou des plantules d'érable sycomore sont présentes dans leur pré. Malgré les outils disponibles sur internet (https://en.wikipedia.org), les propriétaires de chevaux et les vétérinaires éprouvent des difficultés à identifier les différentes espèces d'érables (observations personnelles). Ce phénomène est accentué par les nombreuses descriptions erronées dans la littérature [18]. Un guide de Renaud et al., (2019) est disponible [19], venant en aide aux différents acteurs pour différencier les espèces d'*Acer* communément trouvées dans les pâtures en Europe dans lesquelles des cas ont été déclarés[20].

Le genre érable inclut approximativement 561 espèces [21]. Certaines d'entre elles sont plantées intensivement comme arbres ornementaux pour leurs couleurs automnales. Par conséquent, il n'y a pas seulement des demandes pour différencier les arbres non-toxiques (*Acer platanoides* (érable plane) et *Acer campestre* (érable champêtre)) de l'*Acer pseudoplatanus,* mais des questions sont également posées concernant la toxicité potentielle d'autres espèces d'érables. Près de 50 ans avant la découverte de la cause de la MA, les toxines incriminées avaient été identifiées dans de nombreuses espèces d'érables [3].

Parmi les espèces testées, les espèces suivantes ont obtenu un résultat positif à l'HGA et/ou au MCPG (liste non-exhaustive) : *Acer palmatum, Acer japonicum, Acer macrophyllum, Acer spicatum, Acer saccharinurn,* et *Acer saccharum*. Ces espèces exotiques peuvent être trouvées comme plante

ornementale dans les jardins (Figure 10) et pourraient se propager aux zones voisines [22]. Par conséquent, ces espèces pourraient à l'avenir représenter un risque pour les équidés.

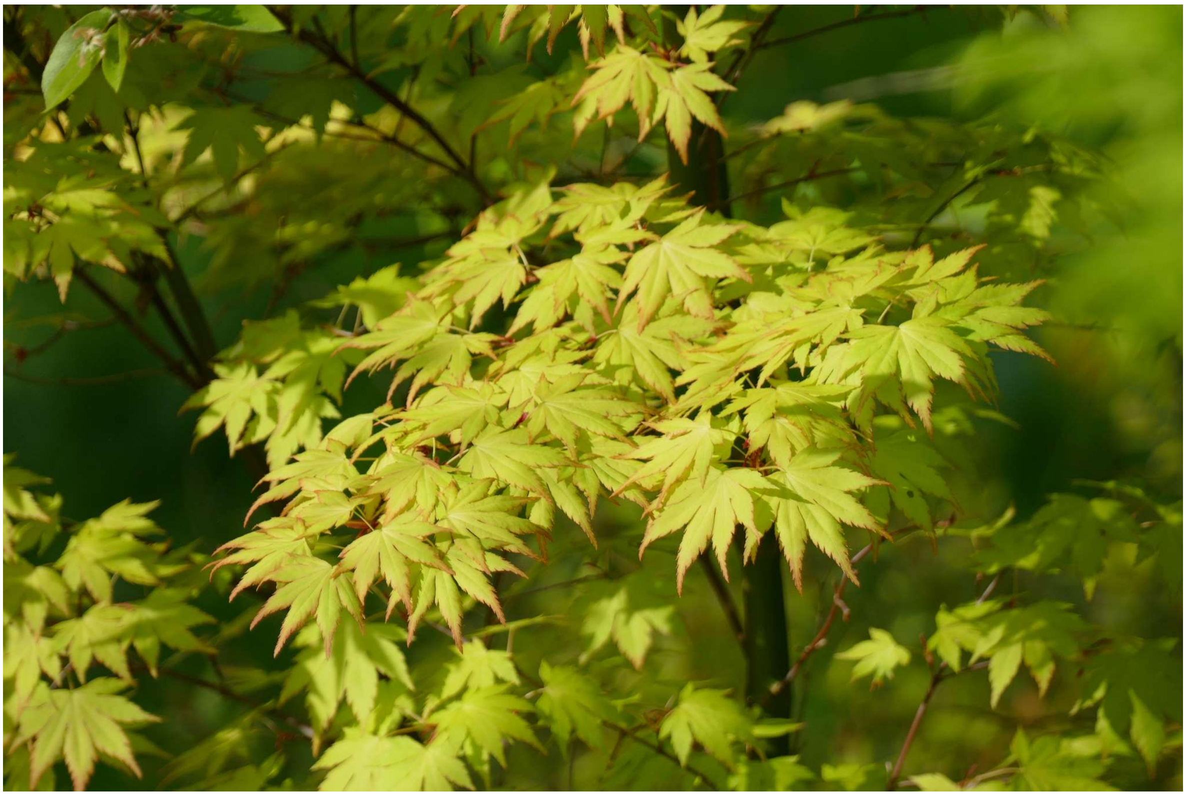

Figure 10. Érable ornemental (*Acer palmatum*)
De nombreuses espèces exotiques d'érables sont utilisées dans les jardins pour leur caractère ornemental. Elles pourraient constituer un risque toxique futur si elles se propageaient dans les pâtures. Crédit photo : D-M. Votion

4.2. FAQ2: « Comment la MA peut-elle être prévenue (au niveau de la pâture)? »

La MA se produit de manière saisonnière avec des séries de cas débutants en automne qui peuvent se prolonger au début de l'hiver. Les séries des cas printaniers, eux, cessent généralement avant l'été. Au niveau de la pâture, le risque peut être diminué (1) en empêchant le contact avec la plante et (2) en favorisant le pâturage sur les prairies les moins à risque en automne et au printemps.

4.2.1. Empêcher le contact avec la plante toxique

De précédentes études épidémiologiques ont identifié comme facteurs de risque de la MA : l'accès à des feuilles mortes entassées en automne (Figure 11), la présence d'arbres et la présence de bois morts [15,17]. Ces observations sont probablement dues à la présence d'*Acer pseudoplatanus* et l'ingestion concomitante de samares et de plantules respectivement à l'automne et au printemps [23–25]. Par conséquent, la reconnaissance d'*Acer pseudoplatanus*, de ses samares (Figure 1) et de ses plantules (Figure 12) est importante. En cas de doute, des expertises réalisées par des professionnels (botanistes et/ou agents des forêts) devraient être commanditées. Récemment, les fleurs de sycomore tombants au sol après de fortes pluies ou des vents violents ont été identifiées comme une probable source additionnelle d'intoxication [26] (Figure 13).

Selon les conditions climatiques, les samares des érables sont capables de se disperser jusqu'à plusieurs centaines de mètres [27]. Par conséquent, la contamination des pâtures avec les samares ou les plantules n'est pas nécessairement liée à la présence d'un arbre sur la pâture. Au début de l'automne et plus particulièrement par vents forts, les samares de sycomore peuvent se disperser ; il est donc recommandé aux propriétaires d'équidés d'identifier les zones contaminées de leur pâture. Le retrait des samares peut contribuer à prévenir la MA [28]. Cependant, lorsque les samares sont

trop abondantes et/ou trop largement dispersées (Figure 14), les zones contaminées devraient être interdites d'accès. Une autre façon de limiter le pâturage à des zones exemptes de samares et/ou de fleurs et/ou de plantules est de créer des parcelles à l'intérieur du pré [26].

L'épandage de fumier et/ou le hersage des prés augmentent le risque de MA [17]. Ces pratiques pourraient favoriser la dispersion des toxines à travers la pâture et par conséquent augmenter le risque d'intoxication.

En ce qui concerne la prévention au niveau des pâturages, il existe un intérêt à éliminer les plantules. Il est à noter qu'après une tonte ou l'application d'un herbicide (Figure 15), les plantules contiennent encore de l'HGA [29]. Ces techniques sont donc inefficaces pour détruire le matériel toxique de la plante.

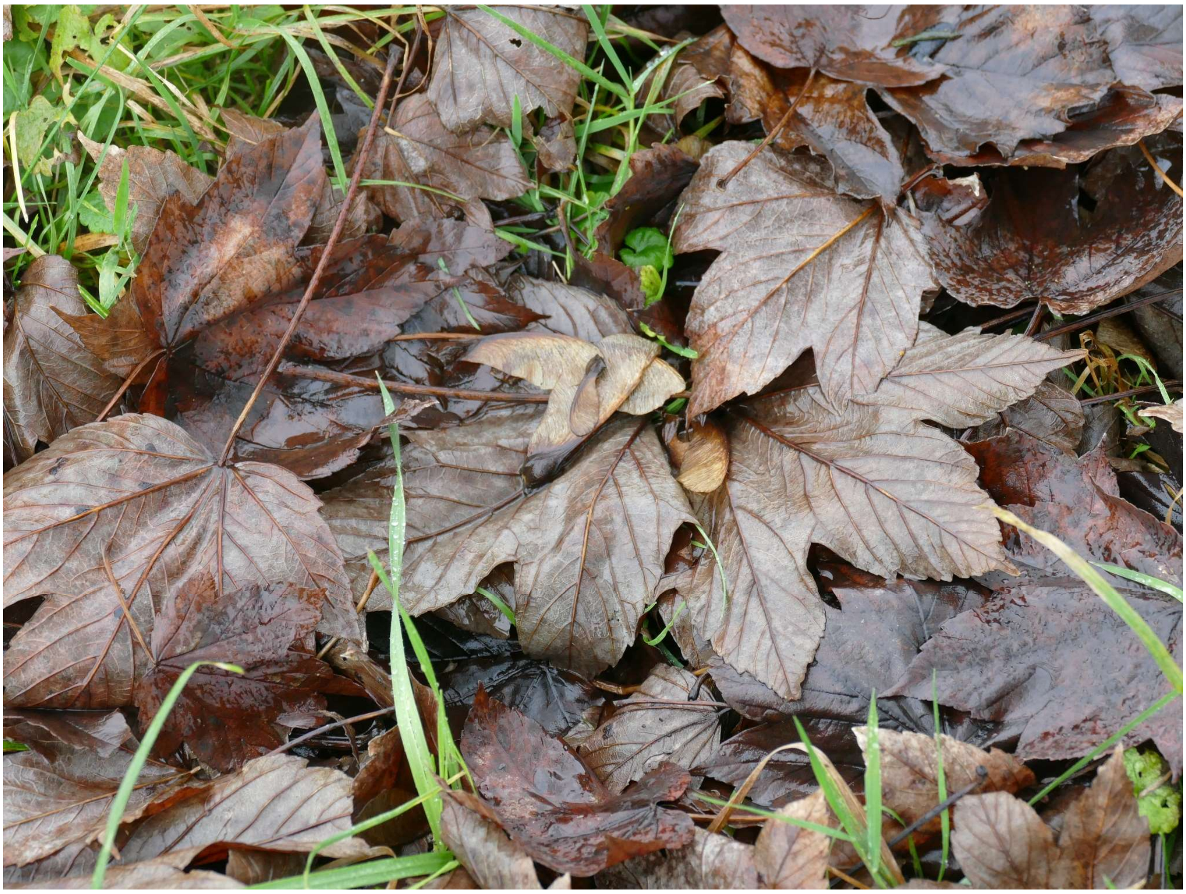

Figure 11. Feuilles mortes accumulées dans une pâture
L'accès à des feuilles mortes entassées en automne a été identifié comme un facteur de risque de la myopathie atypique. Ici, cette observation s'accompagne de la présence de samares de l'*Acer pseudoplatanus* qui sont à l'origine des cas survenant à l'automne. Crédit photo : D-M. Votion

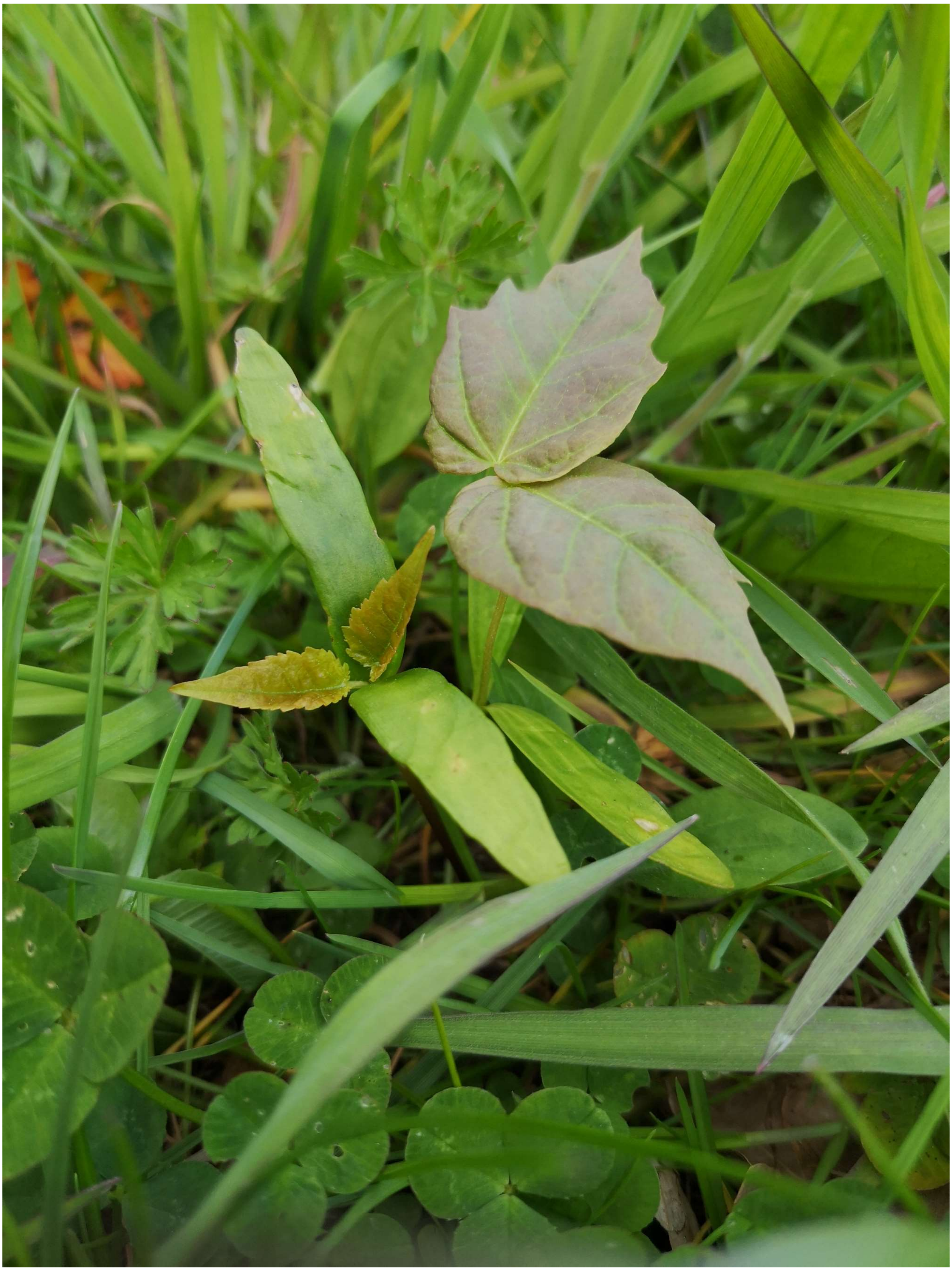

Figure 12. Plantules de l'*Acer pseudoplatanus* (érable sycomore) et de l'*Acer platanoides* (érable plane) Plantules avec leur première paire de feuilles : érable sycomore à gauche et érable plane à droite. Crédit photo : D-M. Votion

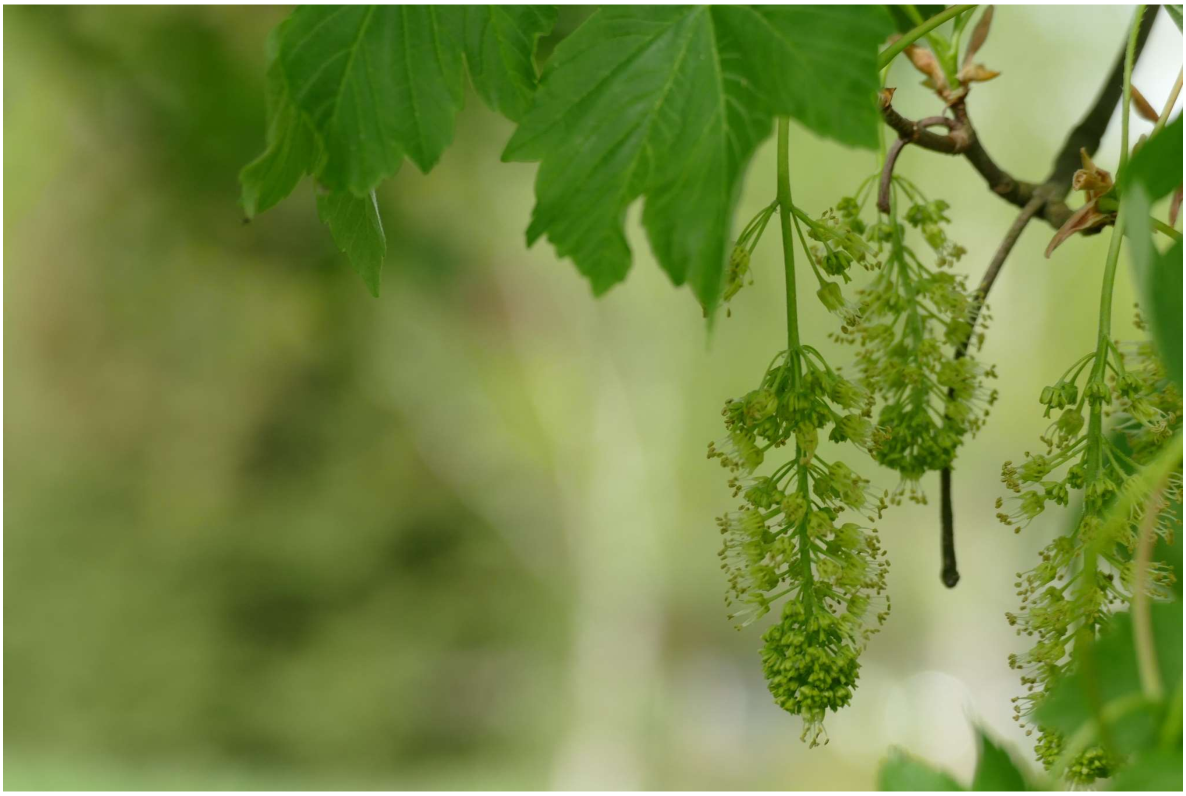

Figure 13. Fleurs de l'*Acer pseudoplatanus* (érable sycomore). Crédit photo : D-M. Votion

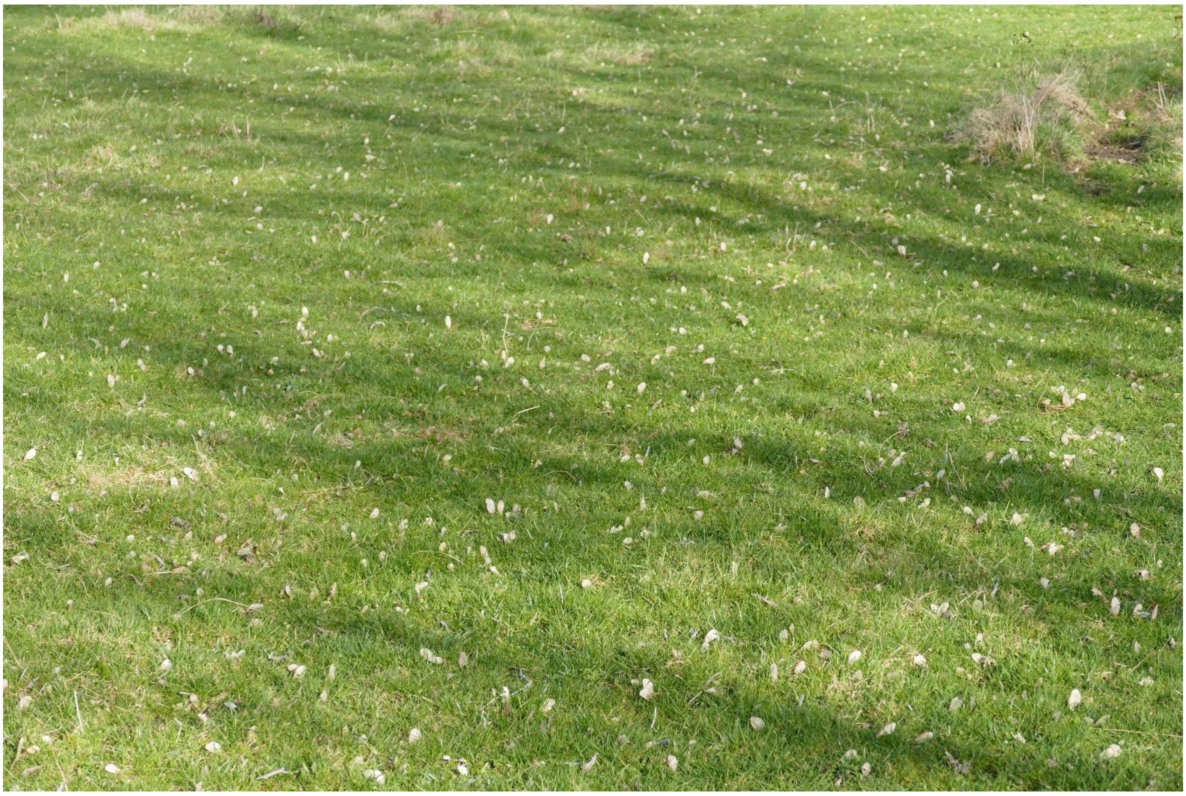

Figure 14. Samares de l'*Acer pseudoplatanus* (érable sycomore) tombées au sol
Lorsque la prairie est largement contaminée, l'accès à celle-ci devrait être proscrit. Crédit photo : D-M. Votion

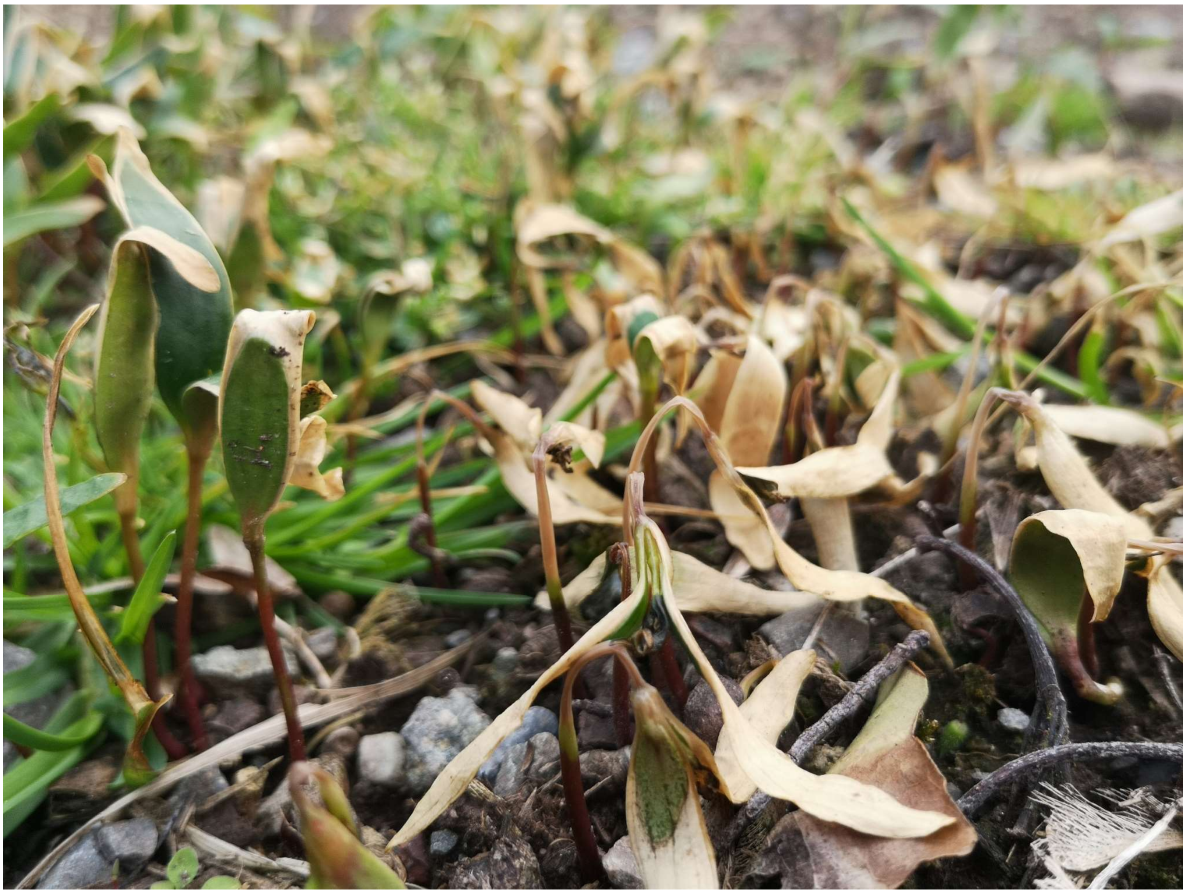

Figure 15. Plantules de l'*Acer pseudoplatanus* (érable sycomore) après traitement phytotoxique Suite au traitement, les plantules flétrissent mais restent toxiques. Crédit photo : D-M. Votion

4.2.2. Utiliser ou créer des pâtures avec peu de risque

Le pâturage permanent a été identifié comme étant un facteur de risque. Ceci est très probablement associé à la surconsommation de l'herbe qui devient rase ce qui inciterait les équidés à ingérer davantage de samares et donc l'agent étiologique [12,15,17]. Notre base de données indique que, dans 64% des cas, la pâture comportait peu ou pas d'herbe (Figure 16). Cette observation est corrélée avec de précédentes études [11]. Une bonne gestion de la pâture (par exemple, des rotations entre pâtures) est conseillée afin d'offrir des pâtures luxuriantes. En effet, un pré fourni en herbe limitera l'ingestion des samares et plantules par les chevaux qui ingèreront davantage d'herbe [11,17].

Les pâtures particulièrement à risque comportent dans leur voisinage des *Acer pseudoplatanus.* Le pâturage sur ces dernières est déconseillé en particulier pendant les saisons à risque (cf. FQ4). En outre, les prairies humides sont aussi à risque et leur pâturage devrait donc être évité [17]. L'HGA est une toxine soluble dans l'eau qui peut passer des plantules à l'eau par contact direct [26,30]; (Figure 17). Cette solubilité pourrait expliquer le risque lié à l'humidité et le facteur de protection lié à l'eau potable fournie par le réseau de distribution. Pour cette raison, seules les prairies ne contenant pas de rivières et/ou d'eau stagnante (Figure 18) devraient être utilisées durant les saisons à risque.

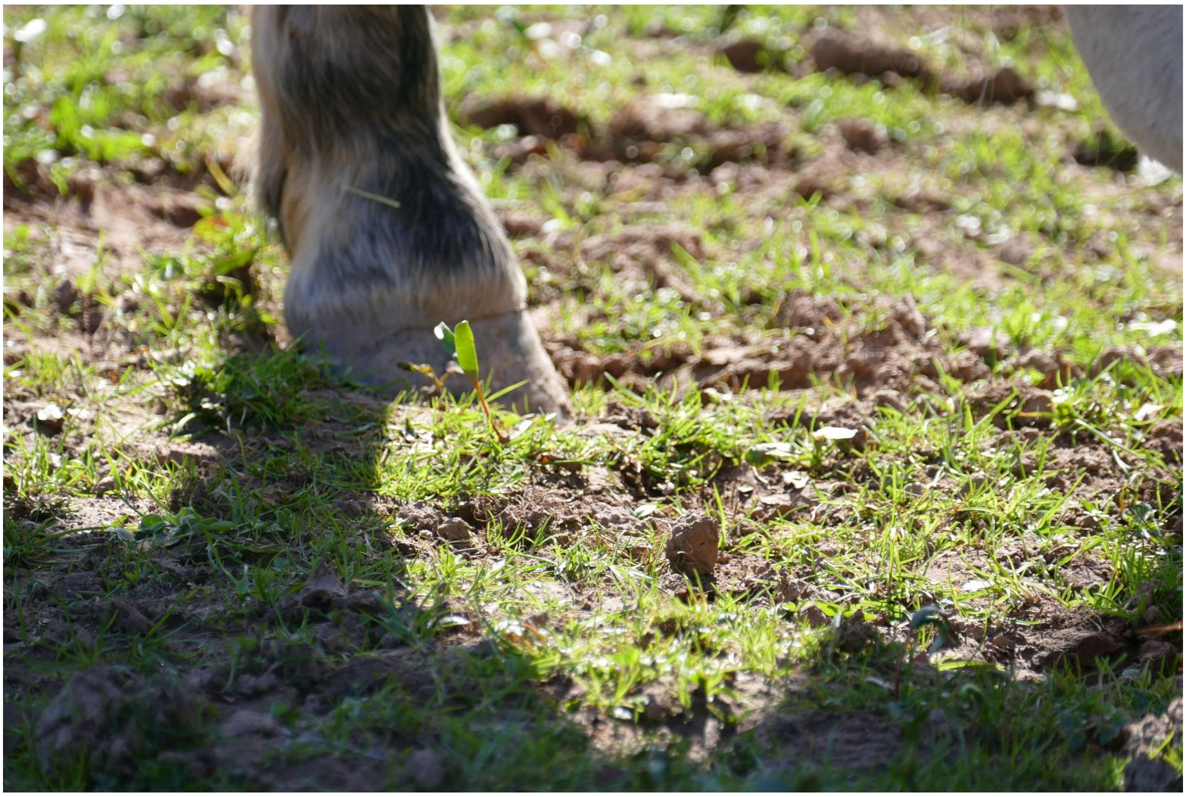

Figure 16. Prairie rase d'un cas belge (printemps 2018). Crédit photo : D-M. Votion

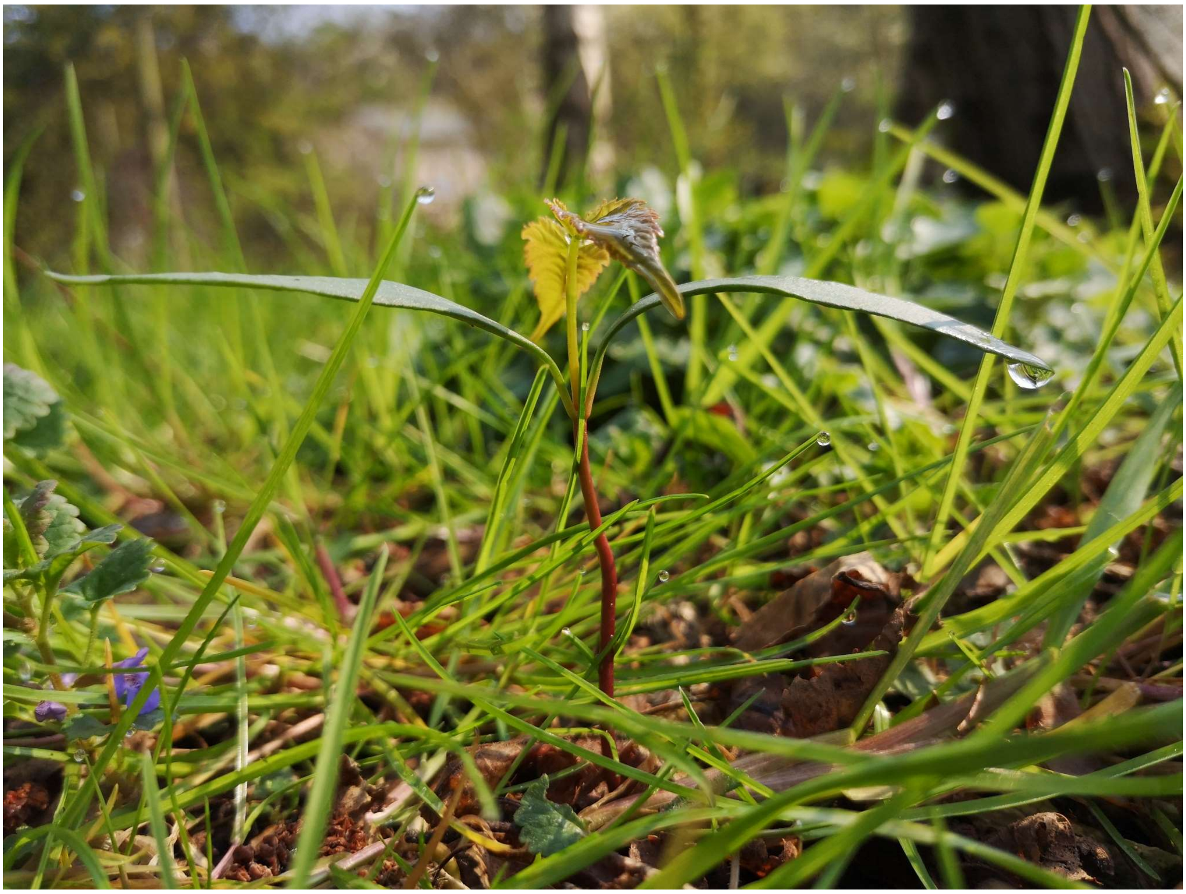

Figure 17. Rosée perlant sur une plantule d'*Acer pseudoplatanus* (érable sycomore)
L'eau qui a percolée sur les plantules contient de l'hypoglycine A [26]. Crédit photo : D-M. Votion

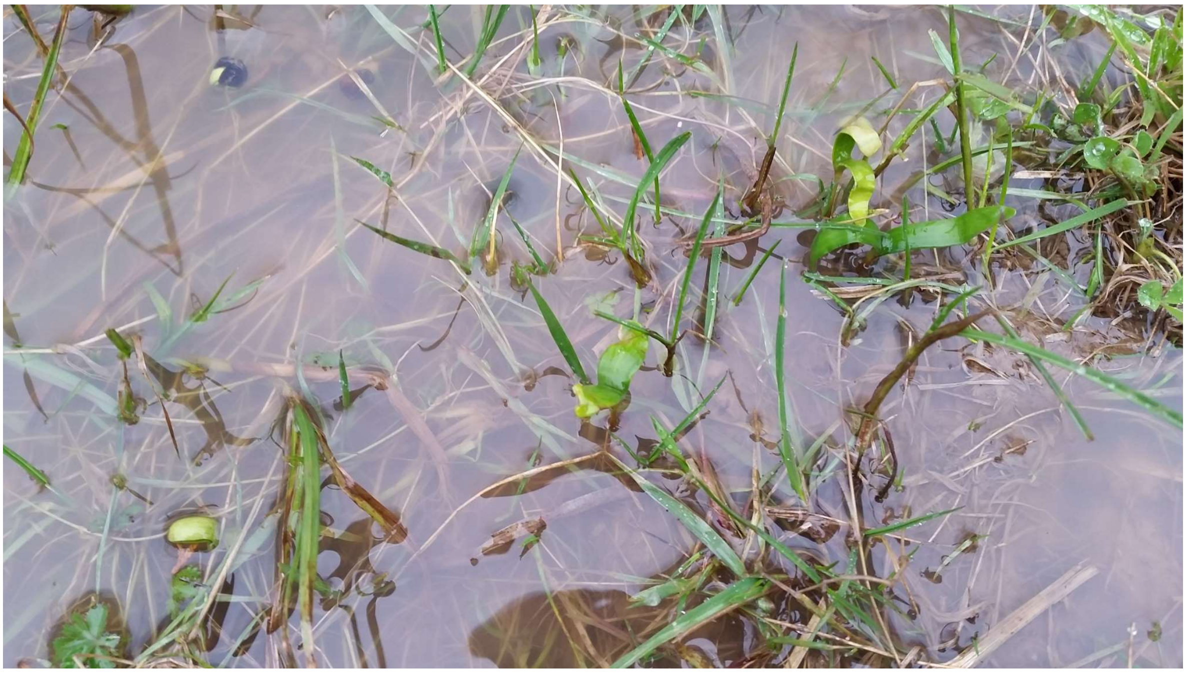

Figure 18. Prairie d'un cas belge (printemps 2015) avec des zones d'eau stagnante d'où émergent des plantules d'*Acer pseudoplatanus* (érable sycomore). Crédit photo : D-M. Votion

4.3. FAQ3: "Comment la MA peut-elle être prévenue (au niveau du cheval) ? »

Tous les équidés peuvent être affectés par la MA y compris les ânes (1.6% des cas) et les zèbres (3 cas dans des parcs zoologiques). Il n'y a pas de facteur de risque associé à une espèce. La première étude épidémiologique a mis en évidence que les chevaux jeunes, spécialement ceux âgés de moins de 3 ans, constituaient le groupe le plus à risque [17]. Plus tard, van Galen et al. (2012) [11] ont observé que tous les groupes d'âges étaient représentés. Nos données suggèrent un changement progressif de la distribution des âges des cas de MA au cours du temps (Figure 8). En 2006, 71% des équidés souffrant de MA étaient âgés de moins de 3 ans alors qu'actuellement, ce groupe ne représente plus que 36% des individus. Il est peu probable que cette évolution puisse être expliquée par l'acquisition d'une immunité face à la toxine étant donné que certains chevaux ayant survécu à la MA ont succombé à un second épisode d'intoxication (données non publiées). Une explication pourrait être que la population à risque augmente en raison d'une augmentation de la pression toxique au cours des années. Tous les équidés doivent donc être considérés comme à risque et ce, qu'importe leur âge. Au niveau de l'animal, le risque peut être diminué par (1) la gestion de l'équidé et (2) d'autres pratiques au niveau alimentation.

4.3.1. Gestion du temps de pâturage

L'intoxication est intimement liée au pâturage comme le rapporte van Galen et al., (2012) avec 98% des chevaux qui ont développé les signes cliniques de la MA alors qu'ils étaient au pré [11]. Nos données confirment cette information avec 99.8% des chevaux en prairie lors de l'apparition des signes d'intoxication. Les cas restant étaient au box depuis moins d'une semaine ce qui implique qu'ils avaient eu la possibilité d'être en contact avec la toxine précédemment. Jusqu'à présent, aucun cas de notre base de données n'a été confirmé par dosage sanguin d'HGA et/ ou de MCPA-carnitine chez des chevaux qui n'avaient pas d'accès à la pâture et/ou au paddock (données non publiées). Il est donc conseillé de maintenir les chevaux au box durant les séries de cas clinques en automne et au printemps si des samares ou des plantules sont, ou peuvent être présentes, dans la pâture [26]. Cependant, garder des chevaux au box, jour et nuit, peut être difficile et contraire à leur bien-être [31,32]. La limitation du temps de pâture à moins de 6 heures par jour est un facteur de protection [15]. Conformément à une étude menée au Royaume-Uni [12], 97.5% des chevaux de notre base de

données passaient *plus* de 6 heures par jour au pré. Cette observation suggère que la durée d'exposition à la toxine est un facteur déterminant du risque de MA. Des conditions météorologiques particulières sont liées aux séries de cas cliniques de MA [9,10,33,34] tandis qu'un pâturage dépendant des conditions climatiques (i.e., rentrer les chevaux au box lorsque des conditions climatiques défavorables sont prévues) réduit le risque de MA [17]. Réduire le temps de pâturage en fonction des conditions météorologiques n'est pas une recommandation mise en œuvre par les propriétaires des cas de notre base de données puisque moins de 1% d'entre eux pratiquaient cette mesure préventive. Nos données renforcent donc la validité de cette mesure préventive.

4.3.2. Approvisionnement en nourriture et en eau

L'apport d'aliments (foin, paille, mélange complet, avoine, orge et/ou maïs) tout au long de l'année diminue le risque de MA [17]. La MA résulte d'un déséquilibre énergétique consécutif à l'intoxication à l'HGA et au MCPG. L'alimentation permet de fournir des substrats (spécialement des hydrates de carbone) qui supportent le métabolisme énergétique et aussi des vitamines et des antioxydants connus pour augmenter le taux de survie [15].
Les enzymes inhibées par l'HGA sont des enzymes dépendantes de la flavine adénine dinucléotide (FAD). Ce cofacteur est originaire de la riboflavine (vitamine B2) et son administration pourrait donc être utile [35]. La luzerne est une source naturelle de riboflavine ainsi que, dans une moindre mesure, le foin [36]. De plus, les équidés bien nourris auront moins tendance à ingérer des samares ou des plantules. Parmi les chevaux recevant des aliments supplémentaires dans leur ration quotidienne (64%; n = 665), nos données indiquent que 61% ont reçu des aliments concentrés (mélange complet, avoine et / ou orge) et 50% avaient accès à un bloc de sel. Cependant, la présence d'une pierre-à-lécher n'a pas empêché ces animaux d'être intoxiqués.

Distribuer du foin en automne a été identifié comme un facteur de risque [17] (et 40% de nos cas recevant des aliments complémentaires ont été nourris uniquement avec du foin). En effet, le foin peut contenir des samares et des plantules (Figures 19) avec une concentration en HGA détectable après plusieurs mois [29] et même des années de stockage [30]. Gonzales et al. (2019) suggèrent que la MA pourrait se produire chez les chevaux au box [29] mais cette hypothèse n'est pas confirmée par nos données car, comme mentionné ci-dessus, pas un seul cas n'a été confirmé grâce à des analyses de sang chez les équidés sans accès au pré et/ou au paddock. Cependant, nous admettons que donner du foin produit à partir de prairies contaminées augmenterait le risque de MA chez les chevaux gardés au pâturage. Il est recommandé de ne pas produire de foin, de préfané et/ ou d'ensilage à partir de prairies jouxtant des érables sycomores [29] ; Figure 20. Fournir du foin en automne reste néanmoins controversé. En effet, cette pratique s'est avérée être un facteur de risque dans une étude cas-témoins [17] mais apparaît comme un facteur protecteur lors de la comparaison des pratiques de gestion des pâturages des cas de MA par rapport aux cas témoins [15]. Ces résultats suggèrent que les fourrages exempts de toxines doivent être donnés *ad libitum*, mais que ceux-ci ne doivent pas être placés sur le sol, ni sous les érables, car ces deux pratiques pourraient augmenter le risque d'ingestion des toxines ; Figure 21.

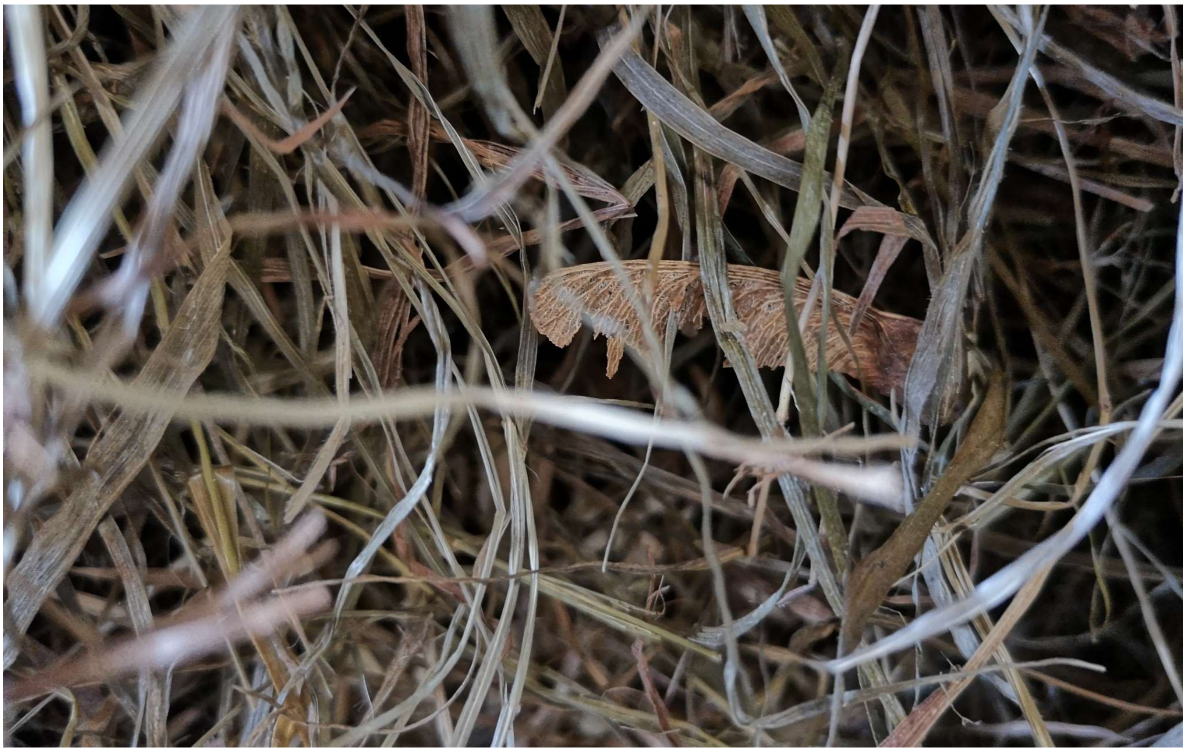

Figure 19. Samare d'*Acer pseudoplatanus* (érable sycomore) dans un ballot de foin. Crédit photo : D-M. Votion

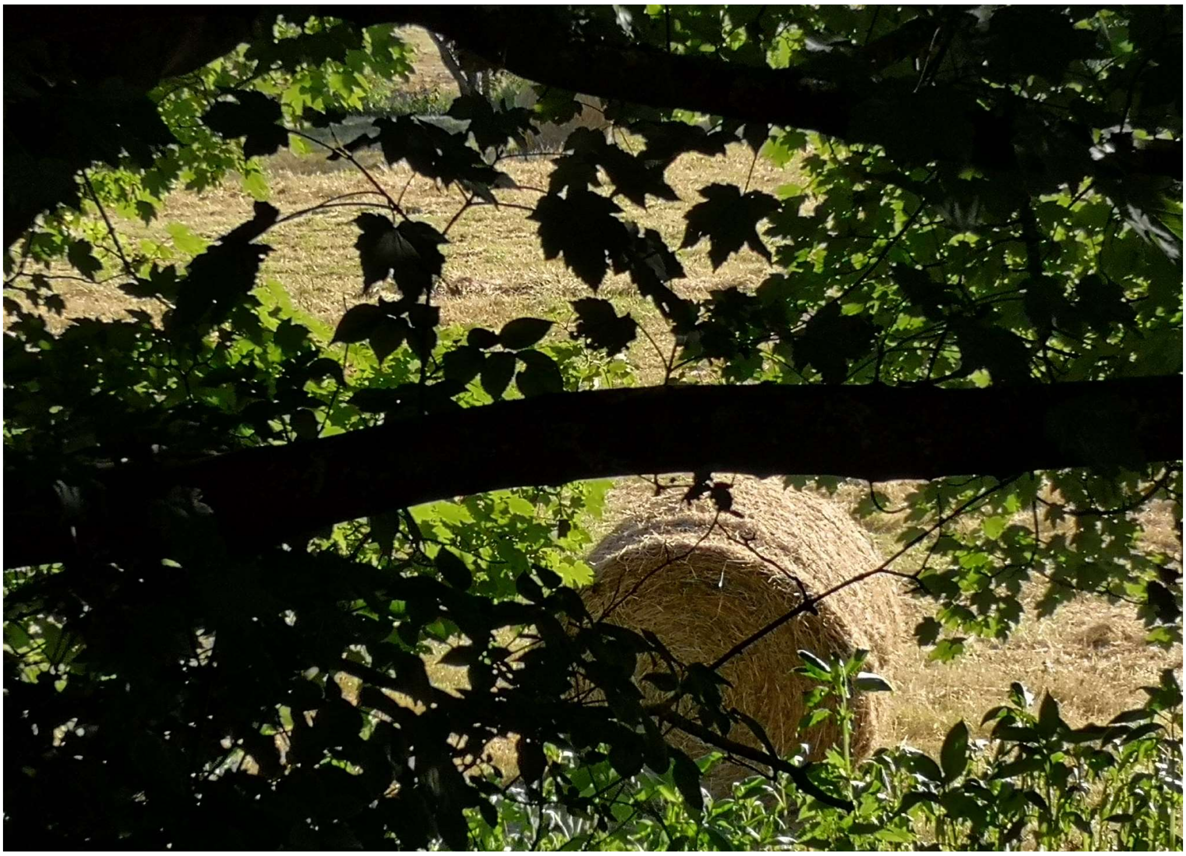

Figure 20. Ballot de foin produit en lisière de forêt
Les fourrages produits en bord de forêt risquent d'être contaminés par des samares et/ ou des plantules si des érables sycomores (*Acer pseudoplatanus*) jouxtent la prairie. Crédit photo : D-M. Votion

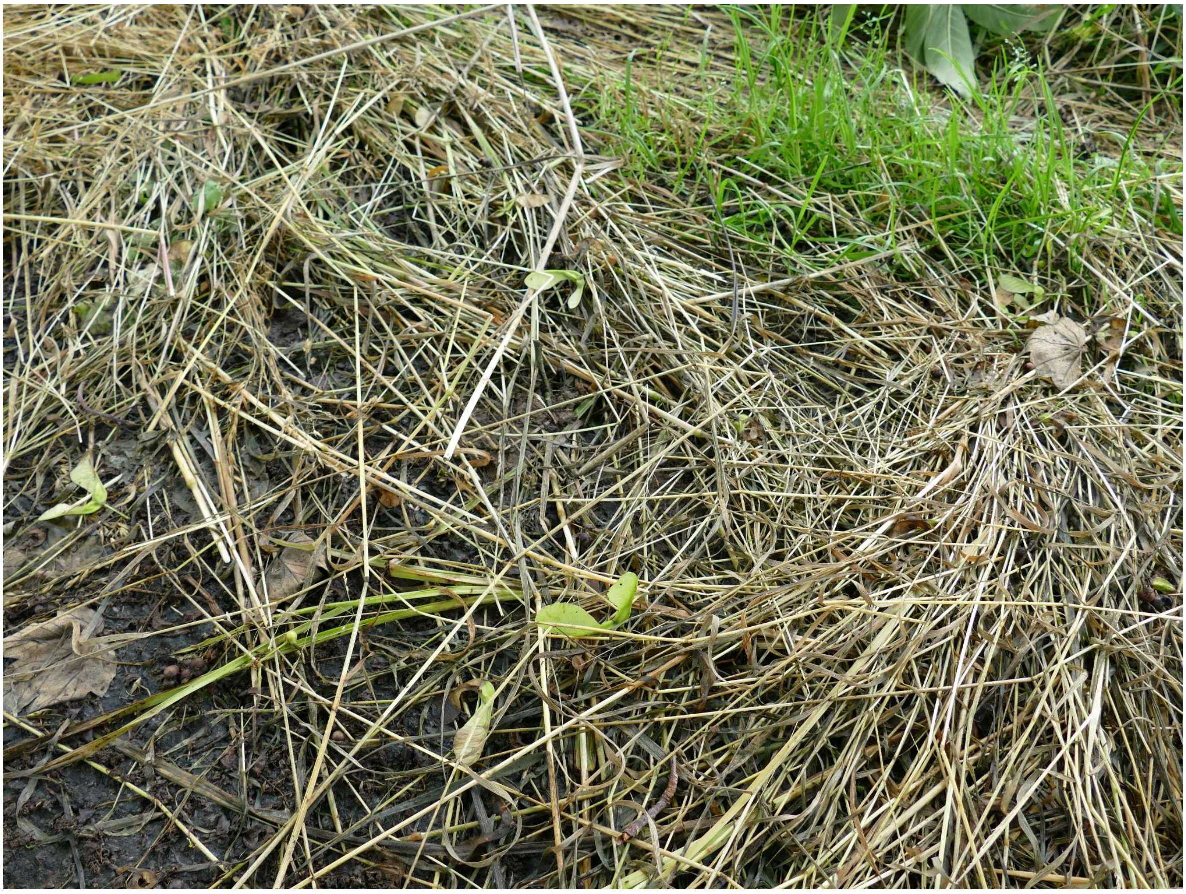

Figure 21. Samares d'érable sycomore (*Acer pseudoplatanus*) tombés sur du foin posé à même le sol. Crédit photo : D-M. Votion

4.3.3. L'eau de boisson

La distribution d'eau via le réseau de distribution [17] est un facteur de protection de la MA. Cette observation suggère que l'eau originaire d'autres sources pourrait être contaminée par la toxine. Cette hypothèse est renforcée par Renaud et al., (2019) [30] qui a montré que de l'HGA est libérée dans l'eau par les fleurs et les samares d'*Acer pseudoplatanus* plongées dans l'eau. D'un autre côté, lorsque l'eau ruissèle sur les fleurs, il n'y a pas d'HGA détectée dans l'eau collectée. Cependant, ces observations peuvent être modulées par le fait que (1) la concentration en HGA peut être inférieur au seuil de détection de la méthode de quantification ou (2) que l'HGA est dégradée par l'eau. À la connaissance des auteurs, il n'existe pas d'étude publiée à propos de la stabilité de l'HGA dans l'eau.

4.4. FAQ4: « Notre pâture est entourée d'érables sycomores, mais il n'y a jamais eu de cas de MA avant. Est-ce que cela signifie que notre cheval est en sécurité dans cette pâture ? »

Nos données et les données des précédentes études épidémiologiques indiquent que des mortalités inexpliquées d'équidés ont eu lieues dans 22% des pâtures antérieurement au cas de MA [11]. En d'autres mots, 80% des cas de MA pâturaient sur des prés sans historique de précédentes mortalités d'équidés (quelle que soit la cause présumée). La MA est une maladie émergente et une prairie entourée de sycomore ne devrait pas être considérée comme étant sans danger pour les chevaux y pâturant même s'il n'y a jamais eu de cas de MA auparavant.

4.5. FAQ5: « Quand le risque de MA commence-t-il et quand se termine-t-il en automne et au printemps ? »

Comme précédemment rapporté, les cas de MA se produisent plus souvent en automne (76%; n = 1801) qu'au printemps (24%; n = 570). Les expressions « automne » et « printemps » ne devraient

pas être prises *stricto senso* car les séries cliniques d'automne continuent au début de l'hiver et certains cas de printemps se produisent après le 21 juin. Il est important de noter que 94% des cas « de printemps » sont survenus entre le 1er mars et le 31 mai et 94% des cas « automnaux » ont été observés entre le 1er octobre et le 31 décembre (Figure 9a,b).

La cause de l'arrêt des cas automnaux n'est pas précisément connue. Avant la découverte de la cause de la MA, l'hypothèse était que le gel était capable de détruire l'agent étiologique car les séries cliniques avaient tendance à s'arrêter après plusieurs jours de gel [10]. À présent que l'HGA est identifiée comme étant la cause de la MA, cette hypothèse peut être réfutée car des investigations en laboratoire ont montré que l'HGA est inaltérée après plusieurs cycles de gel-dégel [37]. Dans notre laboratoire, des samares présentes dans l'environnement ont été collectées de manière hebdomadaire depuis 2016 jusqu'à maintenant et, avec peu d'exception, de l'HGA a toujours été mise en évidence dans les samares de l'érable sycomore. Cependant, les séries cliniques de MA s'arrêtent généralement complètement en hiver et reprennent avec la germination des samares (observations personnelles) ; Figure 22. L'analyse de la concentration en HGA au cours du temps dans des samares tombées au sol révèle une grande variabilité d'un arbre à l'autre et d'une semaine à l'autre empêchant ainsi une interprétation facile de l'évolution de la toxicité. Ces études de terrain ont été compliquées par le fait que les samares récoltées au sol étaient tombées à des moments très différents. Par conséquent, la cause de l'arrêt des séries de cas « automnaux » en hiver n'est pas connue, mais pourrait résulter d'une réduction de l'accessibilité (par exemple, adhérence au sol après la pluie et le gel ; Figure 23) et / ou d'une diminution de la concentration des toxines).

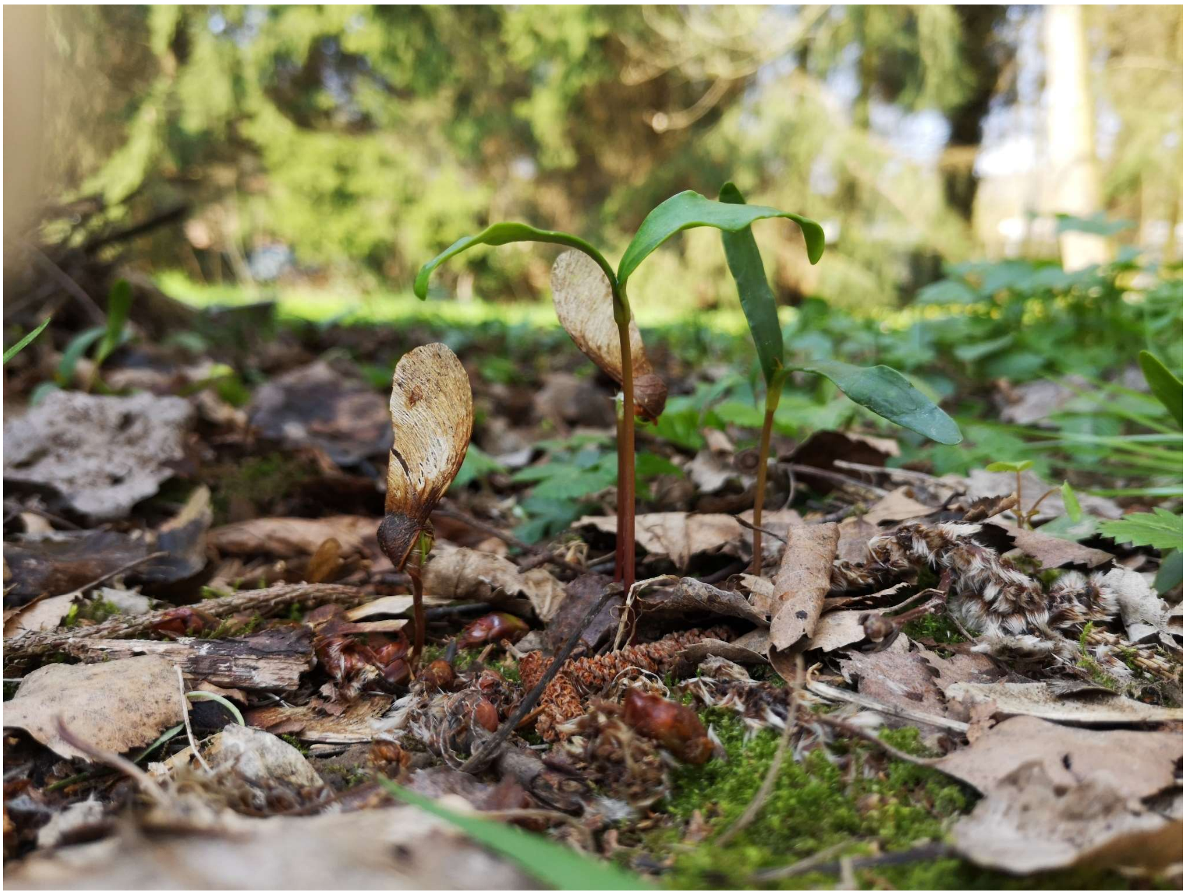

Figure 22. Plantules d'*Acer pseudoplatanus* (érable sycomore) à différents stades de leur germination. Crédit photo : D-M. Votion

Quant au série de cas de printemps, les propriétaires demandent fréquemment si l'arrêt est dû à une perte de toxicité de la plantule. Actuellement, la fin des cas de printemps ne peut pas être expliquée par la disparition de toxicité car les plantules restent toxiques [26]. On suppose cependant que ces cas printaniers cessent à la suite d'une diminution relative du risque d'intoxication lors du

pâturage. Ce risque réduit d'intoxication pourrait résulter (1) d'une prairie luxuriante, (2) de la diminution observée de la toxicité des plantules avec leur croissance [26], (3) d'une diminution de la palatabilité des plantes plus âgées [26], (4) d'une fréquence moindre de conditions météorologiques favorisant la toxicité et (5), d'une disparition naturelle importante des plantules. Concernant ce dernier point, seul un faible pourcentage (<20%) des plantules observées au début du printemps sur des pâturages fortement contaminés sont toujours présentes au début de l'été (données non publiées). Cette observation, ajoutée au fait que la pulvérisation d'herbicide ne réduit pas la concentration en HGA dans les plantules de sycomore [29], remet en question les avantages des traitements phytotoxiques.

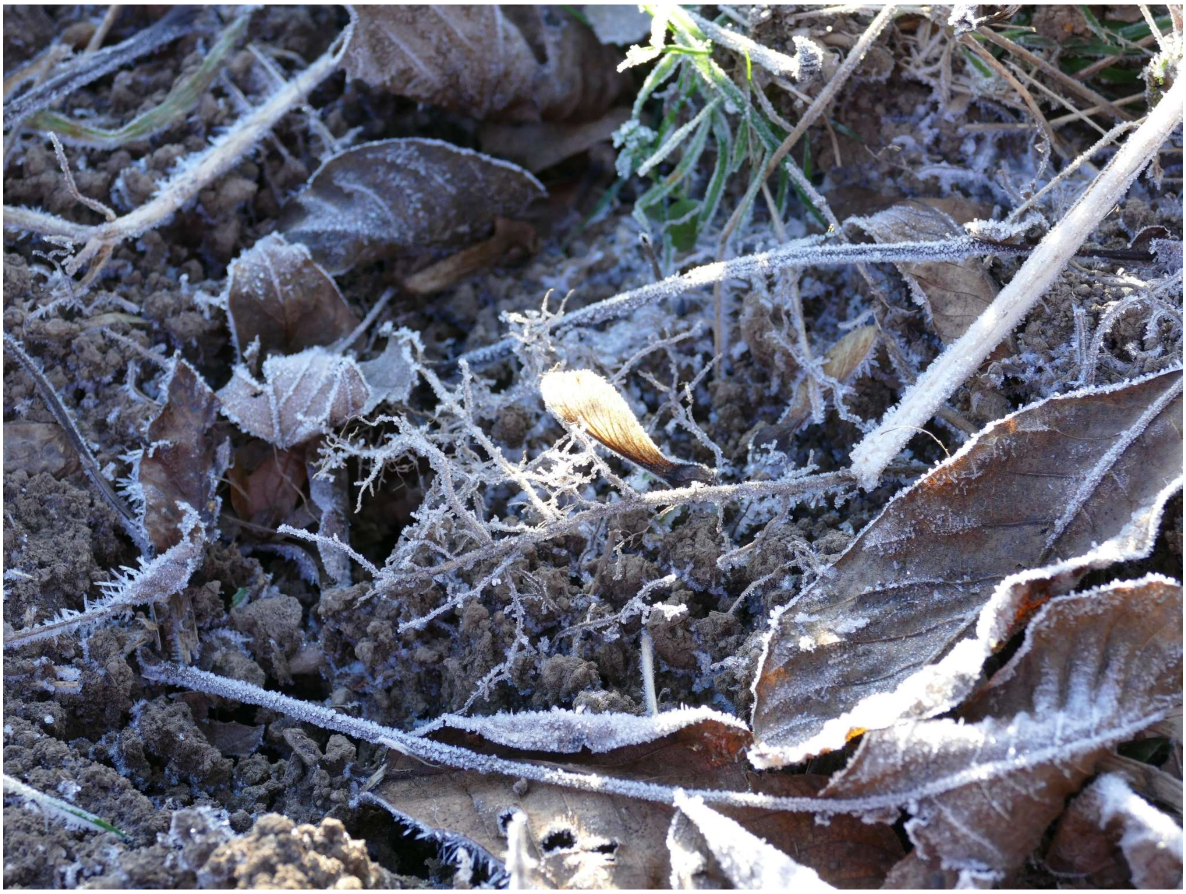

Figure 23. Samare d'*Acer pseudoplatanus* (érable sycomore) sur un sol gelé. Crédit photo : D-M. Votion

5. Conclusions

Étant donné qu'il n'existe pas encore de traitement curatif de la MA, la prévention reste la clé. Le risque de développer la MA résulte de la combinaison de facteurs de risque et de protection. Afin de réduire le risque de MA, il est conseillé d'éviter les prairies humides, le pâturage permanent, le hersage des pâtures et la proximité avec l'érable sycomore. Pendant les périodes à risque, le temps de pâturage devrait être modulé selon les conditions climatiques et limité à moins de 6 heures par jour. Les chevaux pâturant devraient recevoir une alimentation supplémentaire avec, de préférence, de la nourriture contenant de la riboflavine. Lorsque du foin ou de l'ensilage sont distribués, il est nécessaire de faire preuve de prudence en s'assurant que les fourrages sont exempts de toxines. Il est également conseillé de fournir un bloc de sel et de l'eau provenant du réseau de distribution. Il convient de noter que la MA est une maladie émergente et que les équidés de tout âge ainsi que tous les pâturages avec un sycomore à proximité doivent être considérés comme à risque. Ces mesures préventives devraient être mises en œuvre pour une période de 3 mois deux fois par an, à partir de mars pour les « cas de printemps » puis de nouveau en octobre pour éviter les « cas d'automne ».

Comme mentionné précédemment, ce sont les saisons critiques où les samares ou les plantules sont susceptibles d'être présentes dans les pâturages.

Subventions : Cette étude a été soutenue par la Wallonie agriculture SPW (Service public de Wallonie; Belgique), l'« Institut français du cheval et de l'équitation (*Ifce*) » de France et « Les Fonds Spéciaux pour la Recherche (FSR) » de l'Université de Liège (Belgique).

Remerciements : Les auteurs remercient tous les vétérinaires communicants et les propriétaires de chevaux affectés par la MA pour leur collaboration.

Références

1. Votion, D.M.; Van Galen, G.; Sweetman, L.; Boemer, F.; De Tullio, P.; Dopagne, C.; Lefère, L.; Mouithys-Mickalad, A.; Patarin, F.; Rouxhet, S.; et al. Identification of methylenecyclopropyl acetic acid in serum of European horses with atypical myopathy. *Equine Vet. J.* **2014**, *46*, 146–149.
2. Valberg, S.J.; Sponseller, B.T.; Hegeman, A.D.; Earing, J.; Bender, J.B.; Martinson, K.L.; Patterson, S.E.; Sweetman, L. Seasonal pasture myopathy/atypical myopathy in North America associated with ingestion of hypoglycin A within seeds of the box elder tree. *Equine Vet. J.* **2013**, *45*, 419–426.
3. Fowden, L.; Pratt, H.M. Cyclopropylamino acids of the genus Acer: Distribution and biosynthesis. *Phytochemistry* **1973**, *12*, 1677–1681.
4. Bochnia, M.; Sander, J.; Ziegler, J.; Terhardt, M.; Sander, S.; Janzen, N.; Cavalleri, J.M.; Zuraw, A.; Wensch-Dorendorf, M.; Zeyner, A. Detection of MCPG metabolites in horses with atypical myopathy. *PLoS ONE* **2019**, *14*, e0211698.
5. Von Holt, C.; Chang, J.; von Holt, M.; Böhm, H. Metabolism and metabolic effects of hypoglycin. *Biochim. Biophys. Acta* **1964**, *0*, 611–613.
6. Melde, K.; Jackson, S.; Bartlett, K.; Stanley, H.; Sherratt, H.; Ghisla, S. Metabolic consequences of methylenecyclopropylglycine poisoning in rats. *Biochem. J.* **1991**, *274*, 395–400.
7. Westermann, C.M.; de Sain-van der Velden, M.G.M.; van der Kolk, J.H.; Berger, R.; Wijnberg, I.D.; Koeman, J.P.; Wanders, R.J.A.; Lenstra, J.A.; Testerink, N.; Vaandrager, A.B.; et al. Equine biochemical multiple acyl-CoA dehydrogenase deficiency (MADD) as a cause of rhabdomyolysis. *Mol. Genet. Metab.* **2007**, *91*, 362–369.
8. Palencia, P.; Rivero, J.L.L. Short Communications Atypical myopathy in two grazing horses in northern Spain. *Vet. Rec.* **2007**, *161*, 346–348.
9. Finno, C.J.; Valberg, S.J.; Wünschmann, A.; Murphy, M.J. Seasonal pasture myopathy in horses in the midwestern United States: 14 cases (1998–2005). *J. Am. Vet. Med. Assoc.* **2006**, *229*, 1134–1141.
10. Votion, D.M.; Linden, A.; Saegerman, C.; Engels, P.; Erpicum, M.; Thiry, E.; Delguste, C.; Rouxhet, S.; Demoulin, V.; Navet, R.; et al. History and clinical features of atypical myopathy in horses in Belgium (2000–2005). *J. Vet. Intern. Med.* **2007**, *21*, 1380–1391.
11. Van Galen, G.; Marcillaud Pitel, C.; Saegerman, C.; Patarin, F.; Amory, H.; Baily, J.D.; Cassart, D.; Gerber, V.; Hahn, C.; Harris, P.; et al. European outbreaks of atypical myopathy in grazing equids (2006–2009): Spatiotemporal distribution, history and clinical features. *Equine Vet. J.* **2012**, *44*, 614–620.
12. Gonzalez-Medina, S.; Ireland, J.L.; Piercy, R.J.; Newton, J.R.; Votion, D. Equine atypical myopathy in the UK: Epidemiological characteristics of cases reported from 2011 to 2015 and factors associated with survival. *Equine Vet. J.* **2017**, *49*, 746–752
13. Brandt, K.; Brandtt, K.; Hinrrchs, U.; Schulze, C.; Landes, E. Atypische Myoglobinurie der Weidepferde. *Pferdeheilkunde* **1997**, *13*, 27–34.
14. Dunkel, B.; Ryan, A.; Haggett, E.; Knowles, E.J. Atypical myopathy in the South-East of England: Clinicopathological data and outcome in hospitalised horses. *Equine Vet. Educ.* **2018**, 1–6, doi:10.1111/eve.12895.
15. Van Galen, G.; Saegerman, C.; Marcillaud Pitel, C.; Patarin, F.; Amory, H.; Baily, J.D.; Cassart, D.; Gerber, V.; Hahn, C.; Harris, P.; et al. European outbreaks of atypical myopathy in grazing horses (2006–2009): Determination of indicators for risk and prognostic factors. *Equine Vet. J.; Vol.* **2012**, *44*, 621–625.

16. Bunert, C.; Langer, S.; Votion, D.M.; Boemer, F.; Muller, A., Ternes, K. ; Liesegang, A. Atypical myopathy in Pere David's deer (Elaphurus davidianus) associated with ingestion of hypoglycin A. *J. Anim. Sci.*; **2018**, *96*, 3537-3547.
17. Votion, D.M.; Linden, A.; Delguste, C.; Amory, H.; Thiry, E.; Engels, P.; Van Galen, G.; Navet, R.; Sluse, F.; Serteyn, D.; et al. Atypical myopathy in grazing horses: A first exploratory data analysis. *Vet. J.* **2009**, *180*, 77–87.
18. Westermann, C.M.; van Leeuwen, R.; van Raamsdonk, L.W.D.; Mol, H.G.J. "Hypoglycin A Concentrations in Maple Tree Species in the Netherlands and the Occurrence of Atypical Myopathy in Horses. *J. Vet. Intern. Med.* **2016**, *30*, 880–884.
19. Renaud, B.; François, A.-C.; Dopagne, C.; Rouxhet, S.; Gustin, P.; Votion, D. Identification of the Maple Tree Responsible for Atypical Myopathy. Available online: http://hdl.handle.net/2268/242221 (accessed on 12 December 2019).
20. Van Galen, G.; Dopagne, C.; Rouxhet, S.; Pitel, C.; Votion, D. Etiologie de la myopathie atypique : Conditions de toxicité de l'agent causal—Étude préliminaire. In *40ème Journée de la Recherche Equine*; Institut français du cheval et de l'équitation (IFCE): Paris, France, 2014; pp. 101–109.
21. Search Results—The Plant List. Available online: http://www.theplantlist.org/tpl/search?q=Acer&_csv=on (accessed on 12 December 2019).
22. Hulme, P.E.; Bacher, S.; Kenis, M.; Klotz, S.; Kühn, I.; Minchin, D.; Nentwig, W.; Olenin, S.; Panov, V.; Pergl, J.; et al. Grasping at the routes of biological invasions: A framework for integrating pathways into policy. J. *Appl. Ecol.* **2008**, *45*, 403–441.
23. Unger, L.; Nicholson, A.; Jewitt, E.M.; Gerber, V.; Hegeman, A.; Sweetman, L.; Valberg, S. Hypoglycin A Concentrations in Seeds of Acer Pseudoplatanus Trees Growing on Atypical Myopathy-Affected and Control Pastures. *J. Vet. Intern. Med.* **2014**, *28*, 1289–129.
24. Baise, E.; Habyarimana, J.A.; Amory, H.; Boemer, F.; Douny, C.; Gustin, P.; Marcillaud-Pitel, C.; Patarin, F.; Weber, M.; Votion, D.M. Samaras and seedlings of Acer pseudoplatanus are potential sources of hypoglycin A intoxication in atypical myopathy without necessarily inducing clinical signs. *Equine Vet. J.* **2016**, *48*, 414–417.
25. Zuraw, A.; Dietert, K.; Kühnel, S.; Sander, J.; Klopfleisch, R. "Equine atypical myopathy caused by hypoglycin A intoxication associated with ingestion of sycamore maple tree seeds. *Equine Vet. J.* **2016**, *48*, 418–442.
26. Votion, D.M.; Habyarimana, J.A.; Scippo, M.L.; Richard, E.A.; Marcillaud-Pitel, C.; Erpicum, M.; Gustin, P. Potential new sources of hypoglycin A poisoning for equids kept at pasture in spring: A field pilot study. *Vet. Rec.* **2019**, *184*, 740.
27. Katul, G.G.; Porporato, A.; Nathan, R.; Siqueira, M.; Soons, M.B.; Poggi, D.; Horn, H.S.; Levin, S.A. Mechanistic analytical models for long-distance seed dispersal by wind. *Am. Nat.* **2005**, *166*, 368–38.
28. Votion, D. Atypical myopathy: An update. *Practice* **2016**, *38*, 241–246
29. Gonzalez-Medina, S.; Montesso, F.; Chang, Y.-M.; Hyde, C.; Piercy, R.J. Atypical myopathy-associated hypoglycin A toxin remains in Sycamore seedlings despite mowing, herbicidal spraying or storage in hay and silage. *Equine Vet. J.* **2019**, *51*, 701–704.
30. Renaud, B.; Francois, A.C.; Marcillaud-Pitel, C.; Gustin, P.; Votion, D. Myopathie atypique : Les différentes sources d'intoxication. Comment gérer le risque ? In *Journées Sciences et Innovations Équines***,** Institut français du cheval et de l'équitation (Ifce): Saumur, France, 2019; p. 9.
31. Cooper, J.J.; Mason, G.J. The identification of abnormal behaviour and behavioural problems in stabled horses and their relationship to horse welfare: A comparative review. *Equine Vet. J. Suppl.* **1998**, *30*, 5–9.
32. Cooper, J.J.; Albentosa, M.J. Behavioural adaptation in the domestic horse: Potential role of apparently abnormal responses including stereotypic behavior. *Livest. Prod. Sci.* **2005**, *92*, 177–182.
33. Hosie, B.D.; Gould, P.W.; Hunter, A.R.; Low, J.C.; Munro, R.; Wilson, H.C. Acute myopathy in horses at grass in east and south east Scotland. *Vet. Rec.* **1986**, *119*, 444–449.
34. Harris, P.; Whitwell, K. Atypical myoglobinuria alert. *Vet. Rec.* **1990**, *15*, 603.
35. Westermann, C.M.; Dorland, L.; Votion, D.M.; De Sain-van der Velden, M.G.M.; Wijnberg, I.D.; Wanders, R.J.A.; Spliet, W.G.M.; Testerink, N.; Berger, R.; Ruiter, J.P.N.; et al. Acquired multiple Acyl-CoA dehydrogenase deficiency in 10 horses with atypical myopathy. *Neuromuscul. Disord.* **2008**, *18*, 355–364.
36. Rooney, D.K. Applied nutrition. In: Equine Internal Medicine, Eds: D.C. Sellon, S.M. Reed and W.M. Bayly, W.B. Saunders, Philadelphia. **2004**, pp 235-272.

37. Gonzalez-Medina, S.; Hyde, C.; Lovera, I.; Piercy, R.J. Detection of equine atypical myopathy-associated hypoglycin A in plant material: Optimisation and validation of a novel LC-MS based method without derivatisation. PLoS ONE **2018**, 13, 13–15

© 2020 by the authors. Submitted for possible open access publication under the terms and conditions of the Creative Commons Attribution (CC BY) license (http://creativecommons.org/licenses/by/4.0/).
